# Phylogenomics, phenotypic, and functional traits of five novel (Earth-derived) bacterial species isolated from the International Space Station and their prevalence in metagenomes

**DOI:** 10.1038/s41598-023-44172-w

**Published:** 2023-11-06

**Authors:** Anna C. Simpson, Pratyay Sengupta, Flora Zhang, Asif Hameed, Ceth W. Parker, Nitin K. Singh, Georgios Miliotis, Punchappady D. Rekha, Karthik Raman, Christopher E. Mason, Kasthuri Venkateswaran

**Affiliations:** 1 Biotechnology and Planetary Protection Group, Jet Propulsion Laboratory, California Institute of Technology, Pasadena, CA USA; 2 Department of Biotechnology, Bhupat and Jyoti Mehta School of Biosciences, Indian Institute of Technology Madras, Chennai, 600 036 India; 3 Center for Integrative Biology and Systems mEdicine (IBSE), Indian Institute of Technology Madras, Chennai, 600 036 India; 4 Robert Bosch Centre for Data Science and Artificial Intelligence (RBCDSAI), Indian Institute of Technology Madras, Chennai, 600 036 India; 5 Yenepoya Research Centre, Yenepoya (Deemed to be University), Mangalore, 575018 India; 6 Antimicrobial Resistance and Microbial Ecology Group, School of Medicine, University of Galway, Galway, Ireland; 7 Department of Physiology and Biophysics, and the WorldQuant Initiative for Quantitative Prediction, Weill Cornell Medicine, New York, NY USA

**Keywords:** Environmental microbiology, Functional genomics, Genome, Prokaryote

## Abstract

With the advent of long-term human habitation in space and on the moon, understanding how the built environment microbiome of space habitats differs from Earth habitats, and how microbes survive, proliferate and spread in space conditions, is becoming more important. The microbial tracking mission series has been monitoring the microbiome of the International Space Station (ISS) for almost a decade. During this mission series, six unique strains of Gram-stain-positive bacteria, including two spore-forming and three non-spore-forming species, were isolated from the environmental surfaces of the ISS. The analysis of their 16S rRNA gene sequences revealed > 99% similarities with previously described bacterial species. To further explore their phylogenetic affiliation, whole genome sequencing was undertaken. For all strains, the gyrB gene exhibited < 93% similarity with closely related species, which proved effective in categorizing these ISS strains as novel species. Average nucleotide identity and digital DNA–DNA hybridization values, when compared to any known bacterial species, were < 94% and <50% respectively for all species described here. Traditional biochemical tests, fatty acid profiling, polar lipid, and cell wall composition analyses were performed to generate phenotypic characterization of these ISS strains. A study of the shotgun metagenomic reads from the ISS samples, from which the novel species were isolated, showed that only 0.1% of the total reads mapped to the novel species, supporting the idea that these novel species are rare in the ISS environments. In-depth annotation of the genomes unveiled a variety of genes linked to amino acid and derivative synthesis, carbohydrate metabolism, cofactors, vitamins, prosthetic groups, pigments, and protein metabolism. Further analysis of these ISS-isolated organisms revealed that, on average, they contain 46 genes associated with virulence, disease, and defense. The main predicted functions of these genes are: conferring resistance to antibiotics and toxic compounds, and enabling invasion and intracellular resistance. After conducting antiSMASH analysis, it was found that there are roughly 16 cluster types across the six strains, including β-lactone and type III polyketide synthase (T3PKS) clusters. Based on these multi-faceted taxonomic methods, it was concluded that these six ISS strains represent five novel species, which we propose to name as follows: *Arthrobacter burdickii* IIF3SC-B10^T^ (= NRRL B-65660^T^ = DSM 115933^T^), *Leifsonia virtsii* F6_8S_P_1A^T^ (= NRRL B-65661^T^ = DSM 115931^T^), *Leifsonia williamsii* F6_8S_P_1B^T^ (= NRRL B-65662^T^ = DSM 115932^T^), *Paenibacillus vandeheii* F6_3S_P_1C^T^ (= NRRL B-65663^T^ = DSM 115940^T^), and *Sporosarcina highlanderae* F6_3S_P_2^T^ (= NRRL B-65664^T^ = DSM 115943^T^). Identifying and characterizing the genomes and phenotypes of novel microbes found in space habitats, like those explored in this study, is integral for expanding our genomic databases of space-relevant microbes. This approach offers the only reliable method to determine species composition, track microbial dispersion, and anticipate potential threats to human health from monitoring microbes on the surfaces and equipment within space habitats. By unraveling these microbial mysteries, we take a crucial step towards ensuring the safety and success of future space missions.

## Introduction

Multiple low-Earth orbit and lunar orbit space habitats are being planned by governmental and commercial entities as part of the newly revitalized space industry in the 2020s. Cleaning practices and microbial monitoring to ensure crew safety will likely be based on information garnered from studies of the International Space Station (ISS), which is the only space habitat currently in orbit with a long-term history of human habitation. Surfaces of the crew resupply vehicles^1^ and cargo destined to the ISS are cleaned to minimize microbial contamination and protect crew health. Nevertheless, the constant presence of human crew members (approximately six at any given time) on the ISS may lead to the shedding of microorganisms onto its surfaces, potentially causing recontamination and becoming dominant members of the habitat’s microbiome^2^. As a result, ISS surfaces are required to be routinely (once a week) cleaned to maintain a low biomass level (< 10^4^ CFU/100 cm^2^ bacteria and < 100 CFU/100 cm^2^ fungi)^3^.

Despite this rigorous cleaning and maintenance, it has been reported that some resilient microbes, albeit in low numbers, have adapted to microgravity conditions and persist^4^. Examples are the discovery of a novel bacterial genus found repeatedly in metagenomes, which has subsequently been isolated from ISS surfaces during further sampling campaigns^5^, and the isolation of the same clonal strains of *Acinetobacter pittii* from ISS surfaces during different years and sampling campaigns^6^. Characterizing the genomes and adaptation mechanisms of rare or novel species that manage to recolonize ISS surfaces is important because rare species are more likely to have narrow niche breadth, and so are persisting due to adaptive mechanisms to the stresses of radiation, desiccation, and microgravity conditions rather than via generalist strategies. Such microbes are therefore also fertile grounds for biotechnology applications research.

Characterizing novel microbial species on the ISS is crucial for monitoring microbial contamination and protecting astronaut health. The process of whole genome sequencing (WGS) and phenotypic characterization of any novel microbial species found on the ISS helps to identify potential pathogens, as well as to understand potential impact on the spacecraft and its equipment (for example, in biofouling). Easily detecting or identifying a potential microbial threat, whether it be to human health or to the operation of the spacecraft or space station, depends on having an up-to-date, comprehensive database of microbial genomes, their species identity and their phenotypic characteristics (for example, are they known to carry or easily acquire antimicrobial resistance genes, are they UV resistant, what types of surfaces are they more likely to be found on, etc.). This is particularly the case when genetic information about a microbial threat is collected via shotgun metagenomic sequencing rather than by whole genome sequencing of pure culture; in that case, accurate identification relies entirely on an accurate database. The process of characterizing the previously unsequenced cultivable microbiome of space habitats continues to reduce the amount of microbial ‘dark matter’ found in metagenomic sequencing results.

In the ongoing Microbial Tracking (MT) investigation of the ISS^7,8^, out of 510 genomes sequenced, 56 microbial species have been isolated multiple times, representing 27 microbial genera (19 bacteria and 8 fungi). Dominant microbial species include bacterial genera *Staphylococcus, Pseudomonas, Bacillus*, and *Acinetobacter*, yeast genus *Rhodotorula*, and fungal genera *Penicillium*, *Aureobasidium*, and *Aspergillus*^9–25^. In this communication, six strains isolated from various ISS environmental surfaces belonging to five novel bacterial species constituting four different genera (*Arthrobacter, Leifsonia, Paenibacillus* and *Sporosarcina*) are described. Since recent bacterial taxonomy heavily depends on genomic characterization, WGS of these novel species were generated and compared with publicly available genomes of closely related species.

One of the objectives of this study was to establish these six strains as novel species, for which chemotaxonomic, phenotypic, physiological, and phylogenetic (using taxonomic marker genes) analyses were carried out. To discover the phylogenetic placement within their respective genera, we have employed multiple gene analyses and WGS-based phylogenies containing all shared single-copy core genes. The second objective was to quantify the abundance of these recently identified species in the metagenomes of ISS surfaces. Hence, an in-depth analysis was conducted on the metagenomes collected during seven different flight missions, consisting of 106 individual samples, to assess the presence and prevalence of the species. In addition, an attempt was made to generate metagenome assembled genomes (MAGs) of these novel species from the ISS metagenomes. Finally, antiSMASH analysis was performed to identify, annotate, and analyze secondary metabolite biosynthesis gene clusters (BGCs) in the genomes of these novel bacterial species. Specialized metabolites and natural products encoded by these novel bacterial BGCs might provide insight into how different bacterial species thrive and interact in ISS conditions.

## Materials and methods

### Sample collection and isolation

During the MT-1 and MT-2 mission series, samples were collected from the same set of eight surfaces aboard the ISS using pre-packaged and pre-sterilized wipes^7,8^. Upon return to Earth, among other strains belonging to previously defined species, six strains were isolated from both the Advanced Resistive Exercise Device (ARED) platform and crew quarters when grown on blood agar (37 °C for 24 h) or R2A (25 °C for 7 days) media which were unable to be assigned to a known species and were suspected to be novel. Preliminary 16S rRNA gene sequence analysis indicated that strains isolated during Flight 3 (n = 1) from the ARED platform (IIF3SC-B10^T^) and Flight 6 (n = 5) from both the ARED platform (F6_3S_P_1B and F6_3S_P_1C^T^) and crew quarters (F6_8S_P_1A^T^, F6_8S_P_1B^T^ and F6_8S_P_1C) belonged to four different genera: *Arthrobacter*, *Leifsonia*, *Paenibacillus*, and *Sporosarcina*. However, further WGS analysis is required to identify the strains at the species level.

### Light microscopy and SEM

A liquid culture of spore-forming strains was heat shocked (80 °C for 10 min), then plated on Tryptic Soy Agar (TSA) and grown at 30 °C for 5 days to induce endospore formation. Endospore staining with malachite green and safranin was performed using the Schaeffer-Fulton method^26^. Light microscopy and phase contrast images were taken on an Olympus BX53 microscope with an Olympus DP25 camera using Olympus cellSens software.

All strains were grown out on TSA media at 24 °C for 72 h. An isolated colony was fixed in a 4 °C solution of 2.5% glutaraldehyde (Ted Pella Inc., Redding, CA, United States) in 0.1 M sodium cacodylate (NaCaco) (Sigma Aldrich) for an hour. The suspended cells were collected using a vacuum pump and a 0.2 µm Isopore filter membrane (MilliporeSigma, Burlington, MA, United States), and then transferred into a 1.5 ml centrifuge tube. The sample was incubated in 0.1 M NaCaco solution at 4 °C for 10 min and then replaced with fresh 0.1 M NaCaco solution; this washing step was repeated a total of 3 times. The sample then went through dehydration by a step series of increasing IPA and water solutions at 10 min intervals. The series was: 50%, 70%, 80%, 90%, 95% and 100%, with the final 100% rinsing occurring 3 times. The sample was stored at 4 °C in 100% IPA until it was then critically point dried in a Tousimis Automegasamdri 915B critical point dryer (Rockville, MD, United States). Samples were affixed with carbon tape to SEM stubs (Ted Pella Inc.) and then were coated in a ~ 12 nm thick carbon layer by a Leica EM ACE600 Carbon Evaporator (Deerfield, IL, United States). SEM images were collected on a FEI Quanta 200F microscope (Themo Fisher, Waltham, MA, United States) located at the California Institute of Technologies Kavli Nanoscience Institute.

### Biochemical tests and phenotype characterization

Growth temperature and other phenotypic parameters of the tested strains were assessed as follows. Bacterial strains were inoculated in both solid (R2A plates) and liquid (trypticase soy broth (TSB), BD Diagnostics Cat # 257107) media in 15 ml loose-capped centrifuge tubes and grown at temperatures of 4, 15, 25, 30, 37, and 45 °C. Growth on plates and in tubes was monitored daily for 7 days, and incubation was halted if growth was observed. Samples grown at 4 °C and 15 °C were incubated for an additional 4 weeks and 2 weeks, respectively, for final growth assessment. Salt tolerance was determined by inoculating the strains onto R2A plates containing 0–5% added NaCl, as well as agar containing only peptone plus 0 or 1% NaCl and examining growth after 7 days of incubation at 30 °C. Oxidase activity was determined by using OxiDrops™ Liquid Oxidase Reagent (Hardy Diagnostics) on solid culture. Catalase activity was determined by observing effervescence when bacterial colonies were mixed with hydrogen peroxide on a sterile glass slide. Finally, pH tolerance (4 to 10) was tested by adjusting the pH of TSB broth with biological buffers, as described in Xu et al.^27^.

Biochemical tests were performed for each strain using a Gram-positive identification card (Vitek 2 GP ID, bioMérieux) according to manufacturers’ protocol. Briefly, freshly grown colonies were transferred aseptically into a saline (aqueous 0.45% to 0.50% NaCl, pH 4.5 to 7.0) tube to prepare homogenous suspension with a density equivalent to a McFarland No. 0.50 to 0.63 using a calibrated VITEK® 2 DensiCHEK™ Plus. The suspension tube and Vitek 2 GP ID card were placed in the cassette and incubated at 37 °C. Data entry, cassette loading to instrument and retrieval of raw data were done according to the VITEK instrument user manual. Test results were recorded within 10 h of incubation.

Phenotypic fingerprints were generated through GNIII MicroPlate according to BioLog's protocol. Briefly, freshly grown colonies were transferred aseptically into the inoculum solution A (Cat #. 72401; BioLog) to prepare homogenous suspension with a density equivalent to a McFarland No. 0.50. Inoculum was loaded onto BioLog GNIII MicroPlate (100 µl per well) and incubated at 37 °C for 24 h. OmniLog values (A590-A750) were recorded after a minimum 10 h of incubation using MicroPlate reader (FLUO star Omega, BMG Labtech, Germany).

### Chemotaxonomy

To analyze the fatty acid methyl esters (FAME), cells were grown on TSA at 30 °C for 48 h until they reached mid-exponential growth phase. The harvested cell biomass was subjected to saponification, methylation, and extraction^28^ for fatty acid analysis, which was carried out using the Microbial Identification System (MIDI)^29^ with the Aerobe (RTSBA6) database (Sherlock version 6.0) following the standard protocol^30^. A gas chromatograph (Agilent 7890A) with a flame ionization detector was used for FAME analysis, and identification and comparison of the results were made using the MIDI System.

To extract and analyze quinones, polar lipids and peptidoglycans, cells were cultivated on TSA for 3 days at 30 °C. The polar lipids and quinones were extracted and analyzed by two-dimensional thin-layer chromatography (2D-TLC)^31^. To visualize the different classes of polar lipids, the developed TLC plates were treated with 10% (w/v) ethanolic phosphomolybdic acid for total lipids, 0.2% (w/v) ninhydrin in butanol for aminolipids (specific for amino groups), Dittmer and Lester’s Zinzadze reagent for phospholipids (specific for phosphates), and α-naphthol for glycolipids (specific for sugars). Peptidoglycans were extracted and analyzed for the diagnostic amino acids from whole cells^32^.

### DNA extraction and whole genome sequencing

To extract genomic DNA from the novel species, the ZymoBIOMICS DNA MagBead kit was utilized, following the manufacturer's instructions. To prepare the library for WGS, an Illumina Nextera DNA Flex library preparation kit was used as described earlier^10^. Sequencing of prepared libraries was carried out on a NovaSeq 6000 S4 flow cell paired-end 2 × 150-bp platform. The read qualities were evaluated using FastQC v.0.11.7^33^, and subsequenly quality filtered, trimmed and cleared of adapter sequences using fastp v0.20^34^. Draft genomes were assembled using SPAdes v.3.11.1^35^ up to the scaffold level, and the assembly quality was evaluated using QUAST v.5.0.2^36^. The default settings were employed for all steps except for fastp, which included 512 adapters screening.

In addition to Illumina WGS, a secondary round of sequencing was carried out for strains IIF3SC-B10^T^ and F6_8S_P_1B^T^ using Oxford Nanopore sequencing. Additionally, *Sporosarcina thermotolerans* CCUG 53480^ T^, kindly provided by Dr. Edward Moore from the Culture Collection University of Gothenburg (CCUG), Sweden, also underwent WGS with the use of Nanopore MinION (Oxford Nanopore Technologies, Oxford, UK). Nanopore sequencing libraries were prepared using the SQK-RBK114.24 rapid chemistry-based barcoding kit (Oxford Nanopore Technologies, Oxford, UK). Long read sequencing was conducted using an R.10.4.1 flow cell. Base calling was performed via MinKnow/Guppy. Adapters were trimmed using Porechop (v.0.2.4)^37^ and reads were filtered using filtlong v.0.2.1, with parameters *–min_length 1000 –keep_percent 90*. For strains IIF3SC-B10^T^ and F6_8S_P_1B^T^, a hybrid genome assembly was generated using Unicycler (v.0.5.0)^38^. For strain CCUG 53480^T^ a long-read only assembly was generated using the same tool with default settings. Genome assembly quality was assessed using QUAST (v.5.2.0)^36^.

### ANI and dDDH analyses

To elucidate species affiliation of the isolated genomes, we retrieved all validly described and representative genomes of four identified genera from the NCBI database using the command-line tool ‘bit’^39^. We calculated the Average Nucleotide Identity (ANI) and digital DNA–DNA hybridization (dDDH) values to perform pairwise nucleotide-level comparisons. For ANI calculations, we employed FastANI v.1.33, which is a rapid alignment-free computational method, with the novel species as a query against other genomes. To estimate the in-silico DNA–DNA hybridization, we used the recommended formula 2 of the Genome-to-Genome Distance Calculator (GGDC) v.3.0 with the BLAST + alignment tool^40^. To further evaluate the genetic relatedness of the genomes of the genera *Sporosarcina*, ANI based analysis was used. All available genomes on the NCBI GenBank database under the genera *Sporosarcina* (n = 93) were retrieved using ncbi-genome-download (v.0.3.1). An all-vs-all ANI analysis of the genomes was conducted and drawn using ANIclustermap (v.1.2.0).

### Phylogenetic analysis

The 16S rRNA gene sequences of the novel species were extracted from their WGS. Phylogenetic trees were constructed for each genus by incorporating publicly available 16S sequences of all the species within the respective genus. In cases where only the WGS was publicly available, a blast wrapper script was employed to extract the 16S sequence. The trees were rooted using a related species within the same family. The DECIPHER package was used to align and trim the 16S sequences^41^. To build phylogenetic trees, the phangorn package was used^42^ on maximum likelihood with AIC values and 1,000 bootstrap replicates^42^. The trees were visualized using Interactive Tree of Life (iTOL)^43^.

We created a whole genome-based phylogenies to identify closely related species of the isolated genomes. We used GToTree v.1.7.07^44^, a Hidden Markov Model (HMM) based command-line tool which aligns identified single copy genes using Muscle v.3.8 and produces concatenated protein alignment. For the *Arthrobacter* and *Leifsonia* genomes, we used 138 single-copy gene (SCG) sets of *Actinobacteria*, while for the *Paenibacillus* and *Sporosarcina* genomes, we used 119 SCG sets of *Firmicutes*. IQTREE v.2.2.0.3 with ModelFinder-Plus was then used to construct the phylogenetic tree from the protein alignment with 1,000 ultrafast bootstrap replicates^45–47^. We further retrieved 4,552 complete, non-anomalous, representative genomes of bacteria from NCBI Reference Sequence (RefSeq) database and constructed a phylogenetic tree of life along with the novel species using 16 SCG-set as previously described^48^ (data not shown). Genomes containing at least 40% of the total 16 SGC targets were placed in the phylogenetic tree. All the trees were further annotated and visualized using interactive Tree Of Life (iTOL) v.6.7^49^.

### Mapping metagenome sequence reads from ISS to isolated novel species

To investigate the presence of the isolated novel species in environmental samples from ISS, we retrieved paired-end metagenomics reads from two microbial tracking (MT) missions, MT-1 (n = 42) and MT-2 (n = 64), from the NCBI Short Read Archive with project accession PRJNA438545^4^ and PRJNA781277^8^, respectively. Quality filtering of the metagenomes was performed using Trimmomatic v.0.39 with a sliding window of 4 bases and an average quality per base cutoff of 20^50^. We used MetaCompass v.2.0^51^ to perform reference-guided assembly of the aligned metagenome reads against isolated genomes of novel species. We quantified the number of reads that aligned to the isolated genomes and assessed the breadth of the coverage of the assembled reads in each sample. We further tried to bin the contigs using MetaBAT v.2.12.1, but were unable to resolve any MAGs.

### Genome annotation and prediction of secondary-metabolite biosynthetic potential

The gene prediction and annotation of the novel genomes were performed using the Rapid Annotations based on Subsystem Technology (RAST) online server using RAST-tk annotation scheme^52^. The Resistance Gene Identifier (RGI) v6.0.1 web portal which utilizes the CARD v3.2.6 database was used to identify antibiotic resistance genes and markers in the novel species from ISS environments with only “Perfect” and “Strict” matches. Secondary metabolite biosynthetic gene clusters (BGCs) were identified within each novel genomes using antiSMASH v.7.0.0 with a “strict” detection system^53^. The identified BGCs were curated for functional annotation using MIBiG v.3.1 JSON file via an in-house Python script^54^.

## Results

The six strains isolated during this study belonged to five novel species spanning four bacterial genera. Among the six strains, four were non-spore-forming members and two strains formed endospores. Based on 16S rRNA gene similarities, not all strains were identified into a novel bacterial species, but ANI/dDDH analyses allowed them to be placed into five distinct bacterial species. They were: *Arthrobacter burdickii* IIF3SC-B10^T^, *Leifsonia virtsii* F6_8S_P_1A^T^, *Leifsonia williamsii* F6_8S_P_1B^T^, *Paenibacillus vandeheii* F6_3S_P_1C^T^, and *Sporosarcina highlanderae* F6_3S_P_2^T^. In addition, the WGS of *S. thermotolerans* CCUG 53480^T^ was generated and compared with the genome of *S. highlanderae* F6_3S_P_2^T^ to identify the variable, conserved, and distinctive genomic traits.

### Genome characteristics and relatedness indices

Table 1 summarizes the assembly statistics for all six strains. The draft genomes of the novel bacterial species were assembled using high-quality sequences, and their assembly quality varied from a single contig to 49 scaffolds. The non-spore-forming strains had high GC contents, ranging from 68% to 71.4%, whereas spore-forming strains, such as *P. vandeheii* F6_3S_P_1C^T^ (46.1%) and *S. highlanderae* F6_3S_P_2^T^ (41.6%), had low GC content. The total number of predicted genes was 2166 for *S. highlanderae* F6_3S_P_2^T^ and 4861 for *P. vandeheii* F6_3S_P_1C^T^. However, the non-spore-forming bacterial species had ~ 3444 to 3850 coding regions (Table 1). In addition to the novel species, the complete genome of *S. thermotolerans* CCUG 53480^T^ was generated and compared to *S. highlanderae* F6_3S_P_2^T^ strain since both shared > 99% 16S rRNA gene sequence similarities.

**Table 1:** Assembly statistics for novel bacterial species isolated from the ISS and for the type strain of *Sporosarcina thermotolerans.*

Species	Strain #	NCBI Accession #	Isolation location	No. of scaffolds/contigs	Genome size (bp)	N50 (bp)	Average Coverage	G + C Content(%)	Filtered reads used for assembly	Coding sequences
*Arthrobacter burdickii*	IIF3SC-B10^T^	JAROCG000000000	Advanced resistive exercise device (ARED) foot platform, ISS	8	3,949,978	3,120,482	1056	68.0	23,248,163	3,688
*Leifsonia virtsii*	F6_8S_P_1A^T^	JAROCB000000000	Port crew quarters, bump out exterior aft wall, ISS	10	4,198,603	988,071	570	70.5	15,053,671	3,858
*Leifsonia williamsii*	F6_8S_P_1B^T^	JAROCF000000000	Port crew quarters, bump out exterior aft wall, ISS	2	3,889,882	3,847,531	599	71.4	15,606,332	3,444
*Leifsonia williamsii*	F6_8S_P_1C	JARUHI000000000	Port crew quarters, bump out exterior aft wall, ISS	19	3,883,459	550,775	504	71.4	11,128,441	3,439
*Paenibacillus vandeheii*	F6_3S_P_1C^T^	JAROCD000000000	Advanced resistive exercise device (ARED) foot platform, ISS	49	7,035,527	437,229	353	46.1	12,976,930	4,861
*Sporosarcina highlanderae*	F6_3S_P_2^T^	JAROCC000000000	Advanced resistive exercise device (ARED) foot platform, ISS	33	3,397,737	269,276	736	41.6	10,980,322	2,166
*Sporosarcina thermotolerans*	CCUG 53480^T^	CP125968	Human blood	1	3,659,279	N/A	100	41.3	102,168	4,609

Table 2 presents the similarities among closely related members of the novel species based on ANI, dDDH, and two marker genes (16S rRNA and *gyrB*). The 16S rRNA gene sequences of all five novel species described in this study exhibited > 99% similarities to previously established species. However, the gyrB gene sequence similarities of the novel species with the closely related species ranged from 88.6 to 92.8% and could serve as a genetic marker to distinguish the novel species. Moreover, ANI indices (< 95%) and dDDH values (< 70%) fell below the threshold levels of bacterial species identity and confirmed that the examined ISS strains were novel species.

**Table 2:** Whole genome, marker genes sequence similarities, and dDDH values between novel bacterial species and nearest neighbors.

Novel Species (strain number)	Nearest species	WGS Accession #	ANI (%)	dDDH (%)	*gyrB* (%)	16S Accession #	16S (%)
*Arthrobacter burdickii* (IIF3SC-B10^T^)	*Arthrobacter ruber*	GCF_002954225.1	84.6	27.2	88.6	JX949648	99.6
	*Arthrobacter cheniae*	GCF_003602275.1	83.7	25.9	85.9	JX949321	99.9
	*Arthrobacter bussei*	GCF_009377195.2	83.4	25.2	87.4	MN080869	99.9
	*Arthrobacter antioxidans*	GCF_023100725.1	84.1	26.9	88.7	OL471353	99.8
	*Arthrobacter agilis*	GCF_900631605.1	82.9	24.5	87.5	X80748	99.7
*Leifsonia virtsii* (F6_8S_P_1A^T^)	*Leifsonia williamsii* sp. nov	JAROCF000000000	84.3	24.7	91.7	OQ836672	99.2
	*Leifsonia soli*	GCF_013408745.1	86.3	28.3	90.8	EU912483	98.9
	*Leifsonia shinshuensis*	GCF_013410375.1	84.1	24.7	90.6	MH281744	98.9
	*Leifsonia aquatica*	GCF_014190775.1	84.1	25.0	89.4	X77450	97.9
	*Leifsonia naganoensis*	GCF_013410615.1	83.9	24.3	89.6	AB028941	98.0
*Leifsonia williamsii* (F6_8S_P_1B^T^)	*Leifsonia virtsii* sp. nov	JAROCB000000000	84.3	24.7	91.6	OQ836671	99.2
	*Leifsonia aquatica*	GCF_014190775.1	84.5	24.7	90.3	X77450	97.9
	*Leifsonia shinshuensis*	GCF_013410375.1	84.3	25.0	91.1	MH281744	98.9
	*Leifsonia naganoensis*	GCF_013410615.1	84.3	24.7	90.3	AB028941	98.3
	*Leifsonia soli*	GCF_013408745.1	83.9	24.2	91.4	EU912483	98.8
*Paenibacillus vandeheii* (F6_3S_P_1C^T^)	*Paenibacillus xylanivorans*	GCF_001280595.1	92.8	49.3	92.8	KT461879	99.1
	*Paenibacillus tundrae*	GCF_026941245.1	91.0	42.5	95.5	EU558284	98.3
	*Paenibacillus taichungensis*	GCF_013359905.1	90.7	41.5	95.2	EU179327	99.2
	*Paenibacillus pabuli*	GCF_001514495.1	88.4	34.6	94.9	AB045094	99.5
	*Paenibacillus xylanilyticus*	GCF_013359935.1	84.1	26.2	88.5	AY427832	98.3
*Sporosarcina highlanderae* (F6_3S_P_2^T^)	*Sporosarcina thermotolerans*	CP125968	85.3	29.8	87.0	FN298445	99.8
	*Sporosarcina luteola*	GCF_007991495.1	80.5	22.4	81.8	AB473560	99.5
	*Sporosarcina koreensis*	GCF_000826145.2	< 77	20.1	NM	DQ073393	98.8

NM, Not able to measure gyrB gene percent similarity.

### Phylogeny

The comparison of 16S rRNA gene sequences of *A. burdickii* IIF3SC-B10^T^, isolated from the air particles collected from ARED during Flight 2, revealed a high similarity (> 99.6%) to most established *Arthrobacter* species. This suggests that the 16S rRNA gene may not be an adequate marker for distinguishing members within this genus. However, upon analyzing the gyrB gene sequences of this phylogenetic clade, the similarity values were found to be below 85.9%. Furthermore, strain IIF3SC-B10^T^ displayed low values for digital dDDH (24.5%) and ANI indices (82.9%). These findings provide additional evidence supporting its classification as a novel species.

**Figure 1:**
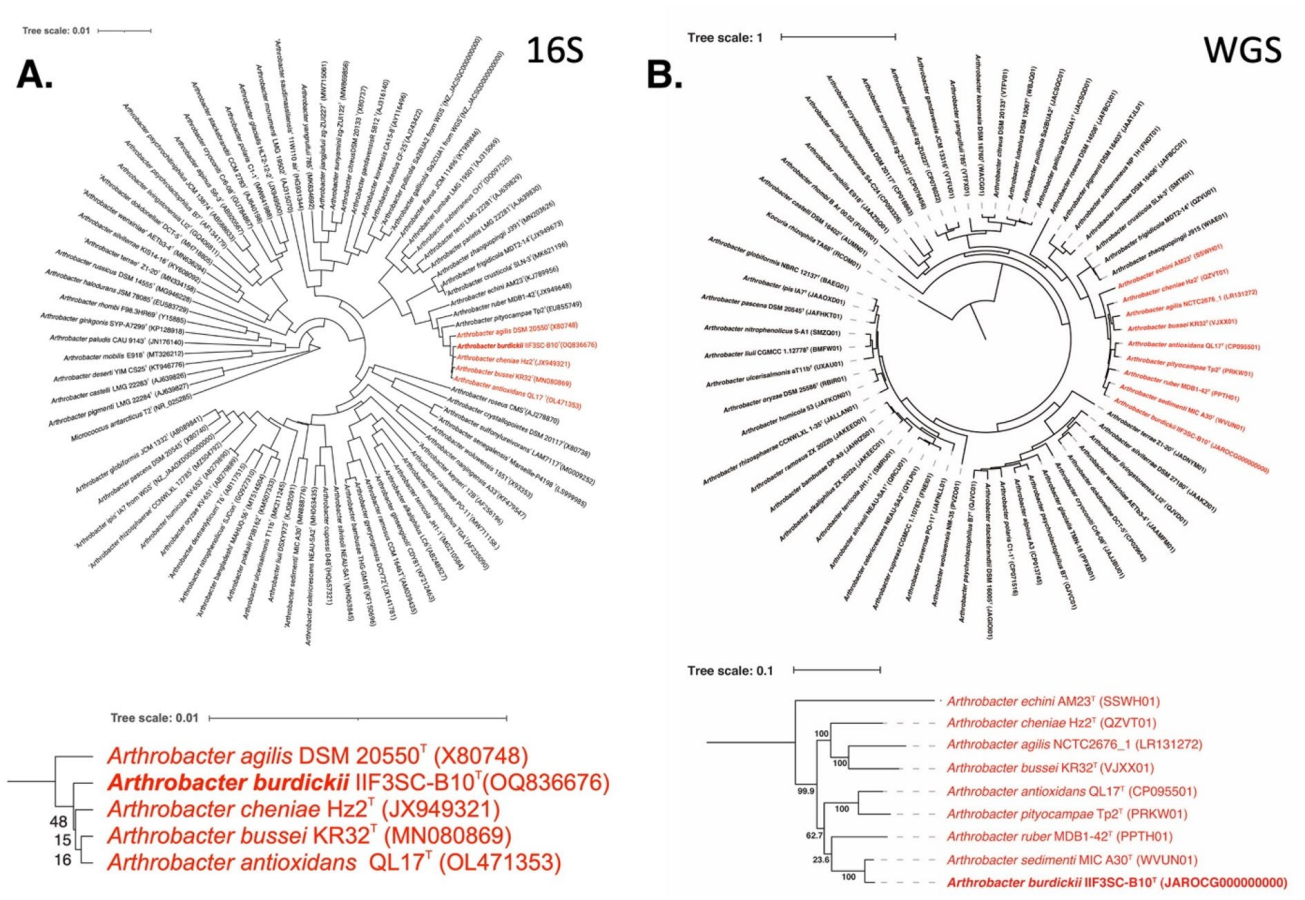
Phylogenetic tree of *Arthrobacter* species including strain IIF3SC-B10^T^ based on (**a**) 16S rRNA genes and (**b**) 138 single-copy core genes of phylum *Actinobacteria*, keeping *Kocuria rhizophila* as an outgroup.

In the 16S rDNA-based phylogenetic tree encompassing all *Arthrobacter* species (Fig. 1A), with *Micrococcus antarcticus* as the outgroup, strain IIF3SC-B10^T^ clustered together with officially named species such as *A. agilis, A. cheniae, A. bussei,* and *A. antioxidans* (Fig. 1A). However, in the WGS-based tree (Fig. 1B), constructed using a concatenated alignment of gene clusters from 59 genomes containing 138 single-copy core genes common to all *Actinobacteria*, *A. burdickii* was found to be distinct from the *A. ruber* and *A. cheniae* clades. Instead, it exhibited closer similarity to the unrecognized species *A. sedimenti* (ANI 93.8%).

**Figure 2:**
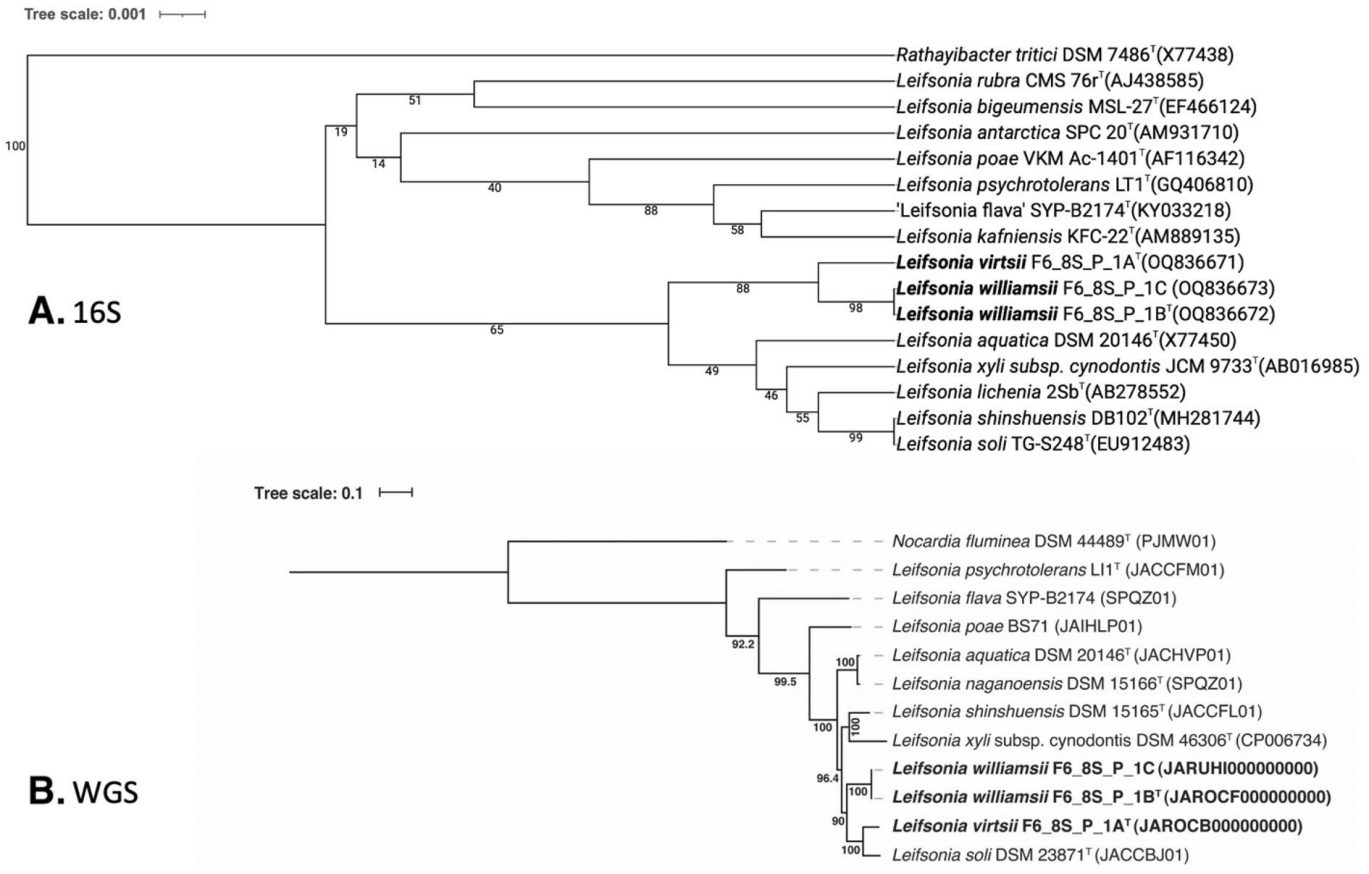
Phylogenetic tree of *Leifsonia* species including strains F6_8S_P_1A^T^, F6_8S_P_1B^T^ and F6_8S_P_1C based on (**a**) 16S rRNA genes and (**b**) 138 single-copy core genes of phylum *Actinobacteria*, keeping *Nocardia fluminea* as an outgroup.

The 16S rRNA gene sequences of *L. virtsii* F6_8S_P_1A^T^, isolated from crew quarters in Flight 6, exhibited > 98.9% similarity to *L. soli* and *L. shinshuensis*, indicating that the 16S rRNA gene is not a suitable marker for distinguishing members of this genus. Upon comparing the *gyr*B gene sequences of the members of this phylogenetic clade, the similarity values were below 91.7%. However, the strain F6_8S_P_1A^T^ exhibited low dDDH values (< 28.3%) and ANI indices (86.3%), providing further evidence that it belongs to a novel species. In the 16S rDNA-based phylogenetic tree of all *Leifsonia* species, with *Rathayibacter tritici* as the outgroup, strain F6_8S_P_1A^T^ was placed within a clade that also included the validly described species *L. aquatica, L. xyli, L. lichenia, L. shinshuensis,* and *L. soli* (Fig. 2A). However, in the WGS-based tree (Fig. 2B), which was constructed using a concatenated alignment of gene clusters of eight available genomes containing a total of 138 single-copy core genes common to all species in the *Actinobacteria*, *L. virtsii* was separated from all these *Leifsonia* species. The next closest member was *L. soli* (ANI 86.3%).

In addition to *L. virtsii*, two strains were identified as *L. williamsii* based on their *gyr*B sequence similarity (91.6%), ANI index (84.3%), and dDDH value (24.7%), which were below the species threshold level. Surprisingly, despite the high 16S rRNA gene sequence similarity between *L. virtsii* and *L. williamsii* (99.2%), the 16S rRNA gene tree (Fig. 2A) placed them in different clades, supported by 88% bootstrap values. Notably, the *L. williamsii* strains were isolated from the same crew quarter location as *L. virtsii*, and they even originated from the same culture plate of R2A medium. Initially, there was a suspicion that they might be clones from the same colony, but further analysis using WGS and *gyr*B sequencing confirmed that they were distinct novel species. In contrast to the 16S rRNA gene phylogeny, the WGS-based tree (Fig. 2B) clearly differentiated *L. williamsii* from *L. virtsii*.

**Figure 3:**
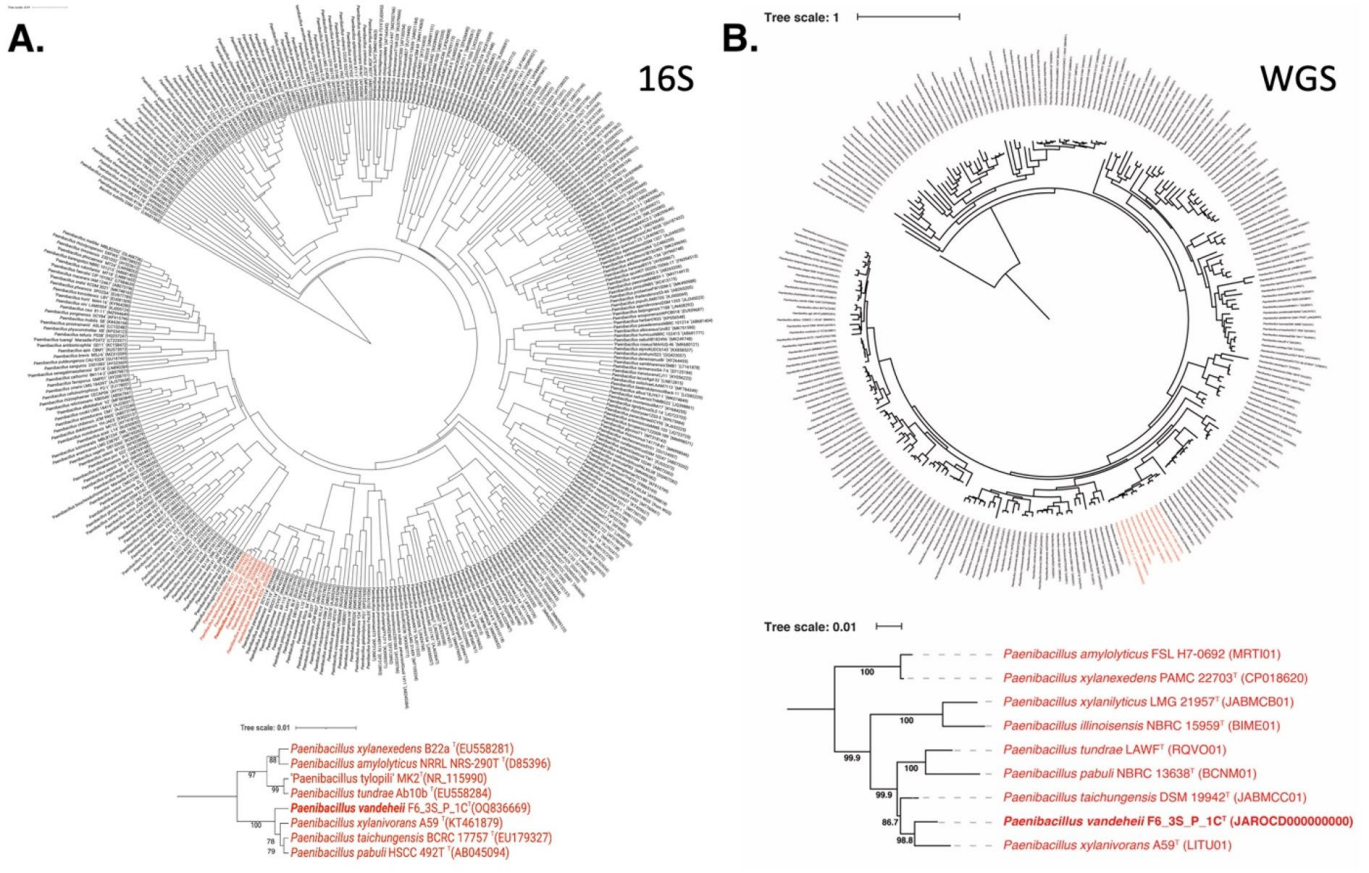
Phylogenetic tree of *Paenibacillus* species including strain F6_3S_P_1C^T^ based on (**a**) 16S rRNA genes and (**b**) 119 single-copy core genes of phylum *Firmicutes*, keeping *Bacillus subtilis* as an outgroup.

The 16S rRNA gene sequences of *P. vandeheii* F6_3S_P_1C^T^, isolated from the ARED’s surface during Flight 6, exhibited 99.5% similarity to *P. pabuli*, indicating that the 16S rRNA gene is not a suitable marker for distinguishing members of this genus. Upon comparing the *gyr*B gene sequences, *P. pabuli* also exhibited 94.9% similarity with *P. vandeheii* F6_3S_P_1C^T^. Since it was established that ~ 95% *gyr*B as cut-off value for species delineation, WGS was performed which showed that ANI index was only 88.4%. Based on low ANI index and dDDH value (34.6%), *P. vandeheii* F6_3S_P_1C^T^ is differentiated from *P. pabuli* and described as a novel species. The 16S rRNA gene-based phylogeny (Fig. 3A) showed that *P. xylanivrans, P. taichungensis,* and *P. paubli* formed a tight clade with > 99% similarities among them. However, in the WGS-based tree (Fig. 3B), which was constructed using a concatenated alignment of gene clusters from 244 genomes containing a total of 119 single-copy core genes common to all species in the *Firmicutes*, *P. vandeheii* was separated from all these *Paenibacillus* species and was found to be closer to the *P. xylanivorans* (ANI 92.8%).

**Figure 4:**
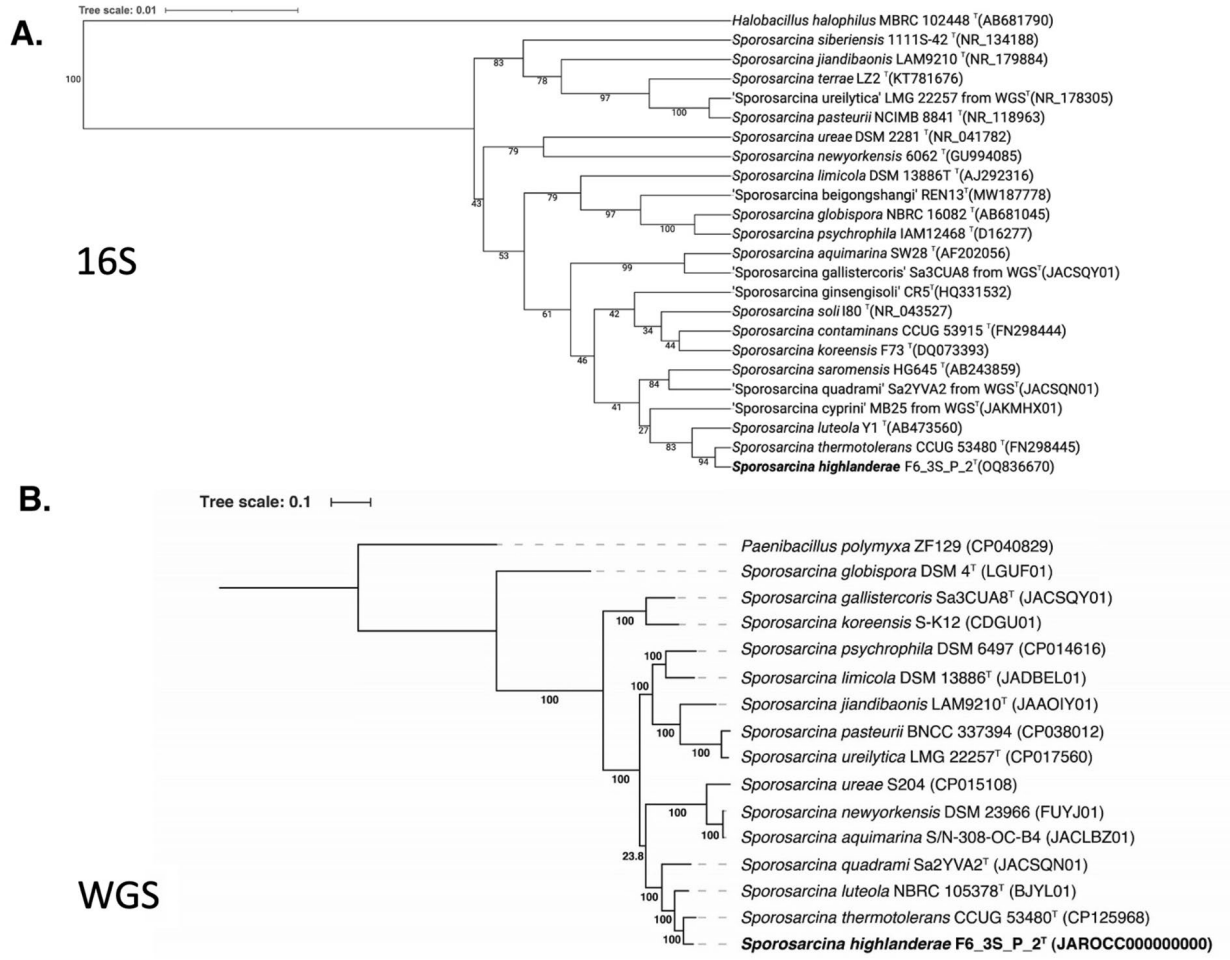
Phylogenetic tree of *Sporosarcina* species including strain F6_3S_P_2^T^ and *Sporosarcina thermotolerans* CCUG 53480^T^ based on (**a**) 16S rRNA genes and (**b**) 119 single-copy core genes of phylum *Firmicutes*, keeping *Paenibacillus polymyxa* as an outgroup.

The strain F6_3S_P_2^T^, another spore-forming bacterium belonging to the genus *Sporosarcina* and isolated from the ARED surface during Flight 6, displayed a 99.8% similarity to *S. thermotolerans* based on the 16S rRNA gene. This finding highlights the difficulty in classifying spore-forming microorganisms using the 16S rRNA gene marker. Hence, the WGS of *S. thermotolerans* CCUG 53480^T^ was needed to identify the phylogenetic position of *S. highlanderae* F6_3S_P_2^T^. Upon comparing the *gyr*B gene sequences, *S. highlanderae* F6_3S_P_2^T^ exhibited an 87.0% similarity with *S. thermotolerans* CCUG 53480^T^. Furthermore, the ANI index between the genomes of F6_3S_P_2^T^ and *S. thermotolerans* CCUG 53480^T^ was only 85.3%. Considering the low ANI index and dDDH value (29.8%), *S. highlanderae* F6_3S_P_2^T^ can be identified as a novel species, distinct from *S. thermotolerans*. In the 16S rRNA gene-based phylogeny (Fig. 4A), *S. thermotolerans, S. luteola,* and *S. saromensis* formed a closely related clade with > 99% similarities among them. However, *S. koreensis* did not cluster within this clade, despite having a 16S rRNA gene similarity with *S. highlanderae*. On the other hand, in the WGS-based tree (Fig. 4B), constructed using a concatenated alignment of gene clusters from 15 genomes containing 119 single-copy core genes common to all species in the *Firmicutes*, *S. highlanderae* was separated from all other *Sporosarcina* species. Instead, it exhibited closer similarity to *S. thermotolerans* (ANI 85.3%).

### Phenotypic characterization

**Figure 5:**
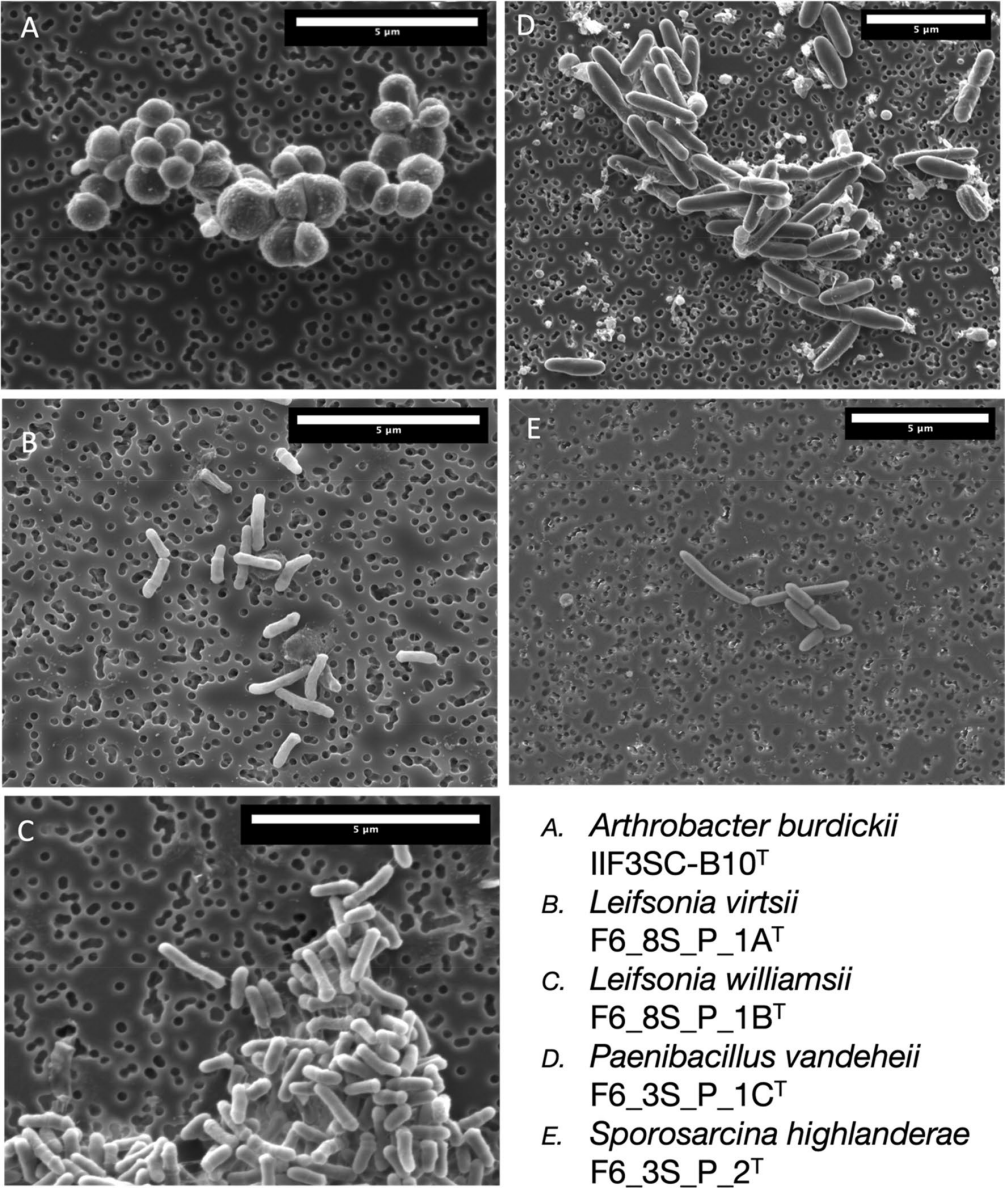
SEM images of novel bacterial species.

The cell size (Fig. 5), colony morphology, biochemical characteristics based on Vitek-2 (Supplemental Table S1) and BioLog GNIII (Supplemental Table S2), fatty acid profiles (Supplemental Table S3) and chemotaxonomic features (Supplemental Figure S1) of all five novel species are presented. *A. burdickii* can be phenotypically differentiated from other closely related *Arthrobacter* species since maltose, trehalose, cellobiose, turanose, and acetoacetic acid were not utilized as sole carbon substrate (Table 3). The *Leifsonia* species did not show any specific phenotypic characteristics that could be used to differentiate them from other closely related *Leifsonia* species; hence, molecular phylogeny is essential (Table 4). *P. vandeheii* was able to grow at 8% NaCl which can be used as discriminative test. Oxidase test was also positive whereas *P. tundrae, P. xylanexedens,* and *P. amylolitus* were negative. In addition, *P. vandeheii* can be differentiated by the utilization of Tween 40, turanose, γ-hydroxybutyric acid, l-malic acid, and l-serine as sole carbon source from *P. taichungensis* and *P. paubli* which are negative (Table 5). *S. highlanderae* could grow at 4% NaCl only, but *S. thermotolerans, S. luteola,* and *S. saromensis* were able to withstand > 7.5% to 10% NaCl concentration (Table 6).

**Table 3:** Differential phenotypic characteristics of *A. burdickii* and closely related species.

Characteristic	*A. burdickii* IIF3SC-B10^T^	*A. cheniae*	*A. bussei*	*A. antioxidans*	*A. agilis*
Maximum tolerable NaCl (%) concentration	5	3	7.5	6.5	4.5
Maximum temperature for growth (°C)	30	30	45	37	35
Oxidase activity	−	−	+	+	−
Maltose	−	+	+	+	+
Trehalose	−	+	+	+	+
Cellobiose	−	+	+	+	+
Turanose	−	+	+	+	+
Acetoacetic acid	−	+	+	+	+
Sucrose	−	+	−	( +)	+
α-d-Glucose	−	+	−	+	+
Stachyose	+	−	+	( +)	−
d-Galactose	+	+	−	+	+
3-Methyl-d-glucose	+	−	( +)	+	+
l-Fucose	+	−	+	+	+
l-Rhamnose	−	+	+	+	−
d-Gluconic acid	+	+	−	+	+
Tween 40	+	+	−	( +)	( +)
Propionic acid	+	+	−	+	−
Alkaline phosphatase	−	−	+	NT	+
Cystine arylamidase	+	+	+	NT	−
Acid phosphatase	−	−	−	NT	+
α-Galactosidase	−	−	+	NT	+

**Table 4:** Differential phenotypic characteristics of *Leifsonia* novel species and closely related species.

Characteristic	*L. virtsii* F6_8S_P_1A^T^	*L. wiliiamsii* F6_8S_P_1B^T^	*L. aquatica*	*L. shinshuensis*	*L. soli*	* L.xyli *subsp.* cynodontis*
Colony color	Yellow	Yellow	Yellow	White	Yellow	Yellow
Oxidase	+	+	+	−	W	−
Growth with 5% NaCl	+	+	+	−	−	−
d-Galactose	+	+	+	−	−	−
d-Glucose	+	+	+	−	+	+
Maltose	+	+	−	−	+	−
d-Mannose	+	+	−	−	+	+
Cellobiose	+	+	+	−	+	−
d-Galactose	+	+	+	W	W	−
d-Glucose	+	+	+	−	+	+
myo-Inositol	−	−	+	+	−	−
Melibiose	+	+	+	+	−	−
d-Rhamnose	+	+	+	W	−	−
Trehalose	+	+	+	+	+	−

**Table 5:** Differential phenotypic characteristics of *P. vandeheii* and closely related species.

Characteristic	*P. vandeheii* F6_3S_P_1C^T^	*P. tundrae*	*P. taichungensis*	*P. pabuli*	*P. xylanexedens*	*P. amylolyticus*
Oxidase	+	−	+	+	−	−
Tolerance to NaCl concentration (%)	8	NT	7	5	NT	NT
Tween 40	+	−	−	−	+	−
d-Gluconic acid	+	−	+	+	+	+
Methyl a-d-glucoside	+	+	−	+	−	+
d-Sorbitol	+	+	−	+	−	+
Turanose	+	+	−	−	−	+
d-Xylose	−	+	+	+	−	+
Acetic Acid	+	+	+	+	−	+
γ-Hydroxybutyric acid	+	−	−	−	+	−
α-Ketoglutaric acid	−	−	−	−	−	−
l-Malic acid	+	+	−	−	+	−
l-Pyroglutamic acid	−	−	−	−	+	−
l-Serine	+	+	−	−	+	−

**Table 6:** Differential phenotypic characteristics of *S. highlanderae* and closely related species.

Characteristic	*S. highlanderae* F6_3S_P_2^T^	*S. thermotolerans*	*S. luteola*	*S. saromensis*
NaCl tolerance (%)	4	10	7.5	9
Urease	−	−	+	+
d-Glucose	+	−	+	−
d-Mannitol	−	−	−	−
d-Fructose	−	NA	+	−

### Chemotaxonomic characterization

The novel actinobacterial species, namely *A. burdickii* IIF3SC-B10^T^, *L. virtsii* F6_8S_P_1A^T^ and *L. williamsii* F6_8S_P_1B^T^, were found to contain diphosphatidylglycerol, phosphatidylglycerol and an unidentified glycolipid as their major polar lipids. Additionally, *A. burdickii* IIF3SC-B10^T^ was found to possess a significant amount of an unidentified phospholipid (PL1), a characteristic that sets it apart from *Leifsonia* species. *P. vandeheii* F6_3S_P_1C^T^ exhibited a complex polar lipid profile, which included phosphatidylglycerol, diphosphatidylglycerol, phosphatidylethanolamine, phosphatidylserine, two unidentified phospholipids and an unidentified aminophospholipid. On the other hand, *S. highlanderae* F6_3S_P_2^T^ was found to contain phosphatidylglycerol, diphosphatidylglycerol, phosphatidylethanolamine two unidentified phospholipids and an identified lipid. The polar lipid profiles of new species are in excellent agreement with the data published earlier for *Arthrobacter*^55^, *Leifsonia*^56^*, Paenibacillus*^57,58^, and *Sporosarcina*^59^.

Based on this polyphasic taxonomy, the five novel species are described, and their detailed phenotypic, FAME profile, chemotaxonomic, and molecular characteristics are given below.

### *Arthrobacter burdickii* sp. nov.

*Arthrobacter burdickii* (bur.dick’i.i. N.L gen. n. *burdickii,* referring to Garry Burdick, an accomplished American space engineer).

Colonies are pink/red-pigmented, convex, round and 0.5 mm in diameter after 3 days of incubation on TSA plate at 25 °C. Cells are coccoid and Gram-stain-positive (approximately 0.7–1.2 μm in diameter with a few that are 1.8 µm in diameter). Growth occurs at 4–37 °C, at pH 5.4–9.3 and in the presence of 0–5% (w/v) NaCl. Positive for catalase and negative for oxidase.

Positive for leucine arylamidase and alanine arylamidase; negative for arginine dihydrolase 1, β-galactosidase, α-glucosidase, ala-phe-pro arylamidase, cyclodextrin, L-aspartate arylamidase, β galactopiranosidase, α-mannosidase, phosphatase, L-proline arylamidase, β-glucuronidase, α-galactosidase, L-pyrrolydonyl-arylamidase, β-glucuronidase, tyrosine arylamidase, and urease.

The major fatty acids (> 10%) are anteiso-C15:0, anteiso-C17:0, and anteiso-C17:1 ω9c. Major polar lipid components are diphosphatidylglycerol, phosphatidylglycerol, an unidentified glycolipid and phospholipids. In addition, two unidentified lipids were found in minor amounts. The predominant menaquinone is MK-9(H2). The peptidoglycan is of A3α type, containing lysine, threonine and alanine.

GC content is 68.0%. The type strain, IIF3SC-B10^T^ (= NRRL B-65660^T^ = DSM 115933^T^) was isolated from the ARED platform aboard the ISS, in 2015; its genome size is ~ 3.9 Mb and available on NCBI, accession number JAROCG000000000.

### *Leifsonia virtsii* sp. nov.

*Leifsonia virtsii* (virts’i.i. N.L gen. n. *virtsii*, named in honor of a NASA astronaut; Terry Virts).

Colonies are circular, convex, smooth, yellow and 1.0–2.8 mm in diameter on TSA medium after 96 h at 25 °C. Cells are aerobic, Gram-stain-positive, motile, short rods, 0.3–0.4 mm wide by 1–2 mm long. Tolerates up to 5% NaCl. Grows at 4–45 °C, with optimum growth at 25 °C, and at pH 5.2–8.6. Positive for oxidase and negative for catalase.

Positive for the oxidation of dextrin, d-maltose, d-trehalose, d-cellobiose, gentiobiosase, sucrose, d-turanose, raffinoase, α-d-Lactose, d-melibiose, d-salicin, N-acetyl-d-glucosamine, α-d-glucose, d-mannose, d-fructose, d-mannitol, glycerol, gelatin, glycyl-l-proline, l-alanine, pectin, d-galacturonic acid, l-galactonic acid lactone, d-gluconic acid, tetrazolium blue, p-hydroxy-phenylacetic acid, methyl puruvate, potassium tellurite, α-hydroxy-butyric acid and sodium butyrate; resistant to rifamycin SV, nalidixic acid and aztreonam. Positive for d-amygdalin, d-xylose, arginine dihydrolase 1, α-glucosidase, leucine arylamidase, α-galactosidase, alanine arylamidase, tyrosine arylamidase, d-galactose, d-mannitol, d-mannose, salicin and sucrose.

Phospholipids detected in major amounts are diphosphatidylglycerol, phosphatidylglycerol and an unidentified glycolipid. Meso 2,6-diminopimelic acid is the diagnostic diamino acid of cell wall.

GC content is 70.5%. The type strain, F6_8S_P_1A^T^ (= NRRL B-65661^T^ = DSM 115931^T^) was isolated from the crew quarters aboard the ISS, in 2018; its genome size is ~ 4.2 Mb and available on NCBI, accession number JAROCB000000000.

### *Leifsonia williamsii* sp. nov.

*Leifsonia williamsii* (wil.li.ams’i.i. N.L. gen. n. *williamsii*, named in honor of a NASA astronaut; Jeffrey Williams).

Colonies are circular, convex, smooth, yellow and 1.0–1.5 mm in diameter on TSA medium after 72 h at 25 °C. Cells are aerobic, Gram-stain-positive, motile, rods, 0.2–0.3 µm in width and 0.8–1.7 µm in length. Tolerates up to 5% NaCl. Grows at 4–45 °C, with optimum growth at 25 °C, and at pH 5.2–9.0. Positive for oxidase and negative for catalase.

Carbon substrate profiles also showed that majority of the carbon substrates and aminoacids are not utilized as sole carbon source. Positive for the oxidation of dextrin, d-maltose, d-trehalose, d-cellobiose, sucrose, d-turanose, raffinose, α-d-lactose, d-melibiose, d-salicin, N-acetyl-d-glucosamine, α-d-glucose, d-mannose, d-fructose, d-mannitol, glycerol, gelatin, glycyl-l-proline, l-alanine, pectin, d-galacturonic acid, l-galactonic acid lactone, d-gluconic acid, p-hydroxy-phenylacetic acid, methyl pyruvate and α-hydroxy-butyric acid; positive for growth at pH 6, 1% NaCl, rifamycin SV, tetrazolium blue, nalidixic acid, potassium tellurite, aztreonam and sodium butyrate.

The major fatty acids (> 10%) are anteiso-C15:0, iso-C16:0, and anteiso-C17:0. Phospholipids detected in major amounts are diphosphatidylglycerol, phosphatidylglycerol and an unidentified glycolipid. In addition, an unidentified lipid was found in minor amounts. The major menaquinone is MK-11. Meso 2,6-diminopimelic acid is the diagnostic diamino acid of cell wall.

GC content is 71.4%. The type strain, F6_8S_P_1B^T^ (= NRRL B-65662^T^ = DSM 115932^T^) was isolated from the crew quarters aboard the ISS, in 2018; its genome size is ~ 3.9 Mb and available on NCBI, accession number JAROCF000000000.

### *Paenibacillus vandeheii* sp. nov.

*Paenibacillus vandeheii* (van. de.hei’i. N.L. gen. n. *vandeheii*, named in honor of a NASA astronaut; Mark Vande Hei).

Cells are Gram-stain-positive, endospore-forming, motile rods (0.6–0.7 µm in width and 2.5–3.5 µm in length). Facultative anaerobe and mesophilic bacterium, with optimum conditions for growth at 30 °C (range 4–37 °C) and pH 7 (range, pH 5.2–9.3), and up to 8% NaCl tolerance. It forms circular and 4 mm in diameter and colonies are beige color on TSA medium. Positive for catalase and oxidase.

Utilize d-raffinose, salicin, saccharose/sucrose, and d-trehalose as sole carbon substrate. Not utilize d-amygdalin, d-xylose, cyclodextrin, d-sorbitol, d-galactose, d-ribose, l-lactate alkalization, lactose, N-acetyl-d-glucosamine, d-maltose, d-mannitol, d-mannose, Methyl-B-d-glucopyranoside, and pullulan. Positive for the production of β-galactosidase, β galactopiranosidase, and α-galactosidase Negative for phosphatidylinositol phospholipase c, arginine dihydrolase 1, α-glucosidase, ala-phe-pro arylamidase, L-aspartate arylamidase, α-mannosidase, phosphatase, leucine arylamidase, L-proline arylamidase, β-glucuronidase, L-pyrrolydonyl-arylamidase, β-glucuronidase, alanine arylamidase, tyrosine arylamidase, urease, and agrinine dihydrolase 2. Resistant to bacitracin and optochin but sensitive to polymixin B and novobiocin.

The major fatty acid is anteiso-C15:0, C16:0, and anteiso-C17:0. The major polar lipids are diphosphatidylglycerol, phosphatidylethanolamine, phosphatidylglycerol, phosphatidylserine, an unidentified phospholipid and an unidentified phosphoaminolipid. In addition, an unidentified phospholipid was found in trace amounts. The predominant menaquinone is MK-7. 2,6-diaminopimelic acid is the major diagnostic diamino acid of the cell wall.

GC content is 46.1%. The type strain, F6_3S_P_1C^T^ (= NRRL B-65663^T^ = DSM 115940^T^) was isolated from the ARED platform aboard the ISS, in 2018; its genome size is ~ 7.04 Mb and available on NCBI, accession number JAROCD000000000.

### *Sporosarcina highlanderae* sp. nov.

*Sporosarcina highlanderae* (high.lan.der’ae. N.L. gen. n. *highlanderae*, referring to Sarah Highlander, an accomplished American molecular biologist).

Cells are Gram-stain-positive, strictly aerobic, motile rods (0.3–0.4 µm in width and 3.3–3.7 µm in length). Spherical endospores are formed in a terminal position. Colonies grown on TSA are circular, convex, beige, and 4 mm in diameter after 5 days at 25 °C. Optimal temperature for growth is 30 °C; growth not at < 10 °C or > 37 °C; pH tolerance is 6.1–9.3. NaCl is not required for growth but can be tolerated up to 4% (w/v). Positive for catalase and oxidase activities.

d-raffinose, salicin, saccharose/sucrose, and d-trehalose, d-amygdalin, d-xylose, cyclodextrin, d-sorbitol, d-galactose, d-ribose, l-lactate alkalization, lactose, N-acetyl-d-glucosamine, d-maltose, d-mannitol, d-mannose, Methyl-B-d-glucopyranoside, and pullulan are not utilized as sole carbon source. Except for the production of tyrosine arylamidase, negative for β-galactosidase, β galactopiranosidase, α-galactosidase, phosphatidylinositol phospholipase c, arginine dihydrolase 1, α-glucosidase, ala-phe-pro arylamidase, L-aspartate arylamidase, α-mannosidase, phosphatase, leucine arylamidase, L-proline arylamidase, β-glucuronidase, L-pyrrolydonyl-arylamidase, β-glucuronidase, alanine arylamidase, urease, and agrinine dihydrolase 2. Resistant to bacitracin and optochin but sensitive to polymixin B and novobiocin.

Major fatty acids are anteiso-C15:0, iso-C15:0, and iso-C14:0. Polar lipid profile contained diphosphatidylglycerol, phosphatidylglycerol, phosphatidylethanolamine, two unidentified phospholipids and an unidentified lipid. MK-7 is the major respiratory quinone. The peptidoglycan type is A4α based on L-Lys-L-Ala-D-Asp.

GC content is 41.6%. The type strain, F6_3S_P_2^T^ (= NRRL B-65664^T^ = DSM 115943^T^) was isolated from the ARED platform aboard the ISS, in 2018; its genome is ~ 3.4 Mb and available on NCBI, accession number JAROCC000000000.

### Abundance of novel species in ISS metagenomes

**Figure 6:**
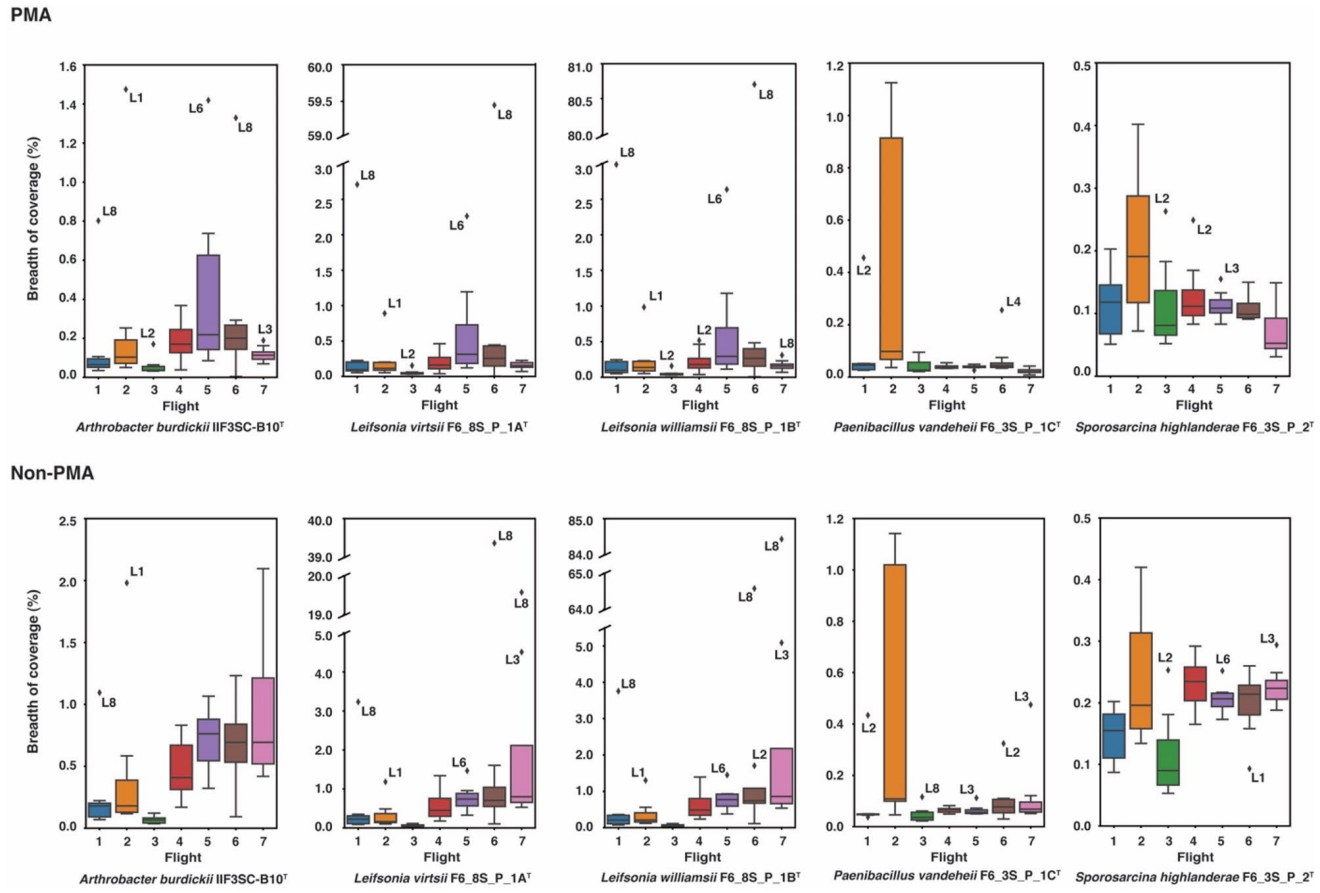
Box plots showing breadth of coverage of the consensus genome constructed from mapped reads aligned to the novel species (percent coverage was less than 1% in all cases). Due to high variations in the breadth of coverages of *Leifsonia* genomes, a broken Y-axis was used. The reads were collected from eight locations on the ISS across seven separate time points/flights as part of the Microbial Tracking project(s).

We conducted an analysis of metagenomic reads obtained from two microbial tracking (MT) missions, encompassing seven flights across eight locations on the ISS, with the objective of identifying novel microbial species and potentially retrieving MAGs. To assess the presence of viable and intact cells of the novel species, we utilized propidium monoazide (PMA) treatment on the samples as previously described^7,8^. Our findings revealed that the majority of the metagenomes had less than 0.1% of their total reads mapped to the novel species. Among all the species analyzed, *P. vandeheii* F6_3S_P_1C^T^ exhibited the highest mapping, with a maximum of 1.05% of total reads from a sample collected during Flight 2 near the port crew quarters (location 8) during MT-1. Therefore, we can conclude that none of these novel species are dominant in the ISS. Considering the limited proportion of reads mapped to the novel species, we proceeded to perform read assembly to explore the breadth of coverage against the isolated genomes. Interestingly, reads mapped to *L. virtsii* F6_8S_P_1A^T^ and *L. williamsii* F6_8S_P_1B^T^ from Flight 6 at location 8 exhibited a breadth of coverage of 59.4% and 80.7% respectively, despite representing a small fraction of the total reads (Fig. 6). However, apart from these cases, the overall breadth of coverage for the genomes was quite low, averaging at 0.21% hence, no MAG was generated. We also analyzed the PMA untreated samples and observed the similar pattern in the distribution of the breadth of coverages. Additionally, we noticed 84.4% breadth of coverage for *L. williamsii* F6_8S_P_1B^T^ during Flight 7 at location 8 from where the strain was isolated. Despite the presence of a high breadth of coverage for *Leifsonia* genomes across multiple samples, we were unable to obtain MAGs due to the failure of achieving the minimum coverage depth threshold of 4X.

### Functional characterization of the novel species

To investigate the genetic characteristics of the six novel strains, we performed a comprehensive genome annotation using RAST-tk (Table 7; Supplemental Table S4). The subsystem mapping results are as follows: *A. burdickii* IIF3SC-B10^T^ (283 subsystems), *L. virtsii* F6_8S_P_1A^T^ (273 subsystems), *L. williamsii* F6_8S_P_1B^T^ (262 subsystems), *L. williamsii* F6_8S_P_1C^T^ (262 subsystems), *P. vandeheii* F6_3S_P_1C^T^ (324 subsystems), and *S. highlanderae* F6_3S_P_2^T^ (307 subsystems). Among the annotated subsystems, the top categories based on average gene counts included amino acids and derivatives (260 genes), carbohydrate metabolism (232 genes), cofactors, vitamins, prosthetic groups, pigments (136 genes), and protein metabolism (171 genes).

**Table 7:** Antimicrobial and stress resistance genes detected in novel ISS species.

Category	Gene	*A. burdickii*	*L. virtsii*	*L. williamsii* F6_8S_P_1B^T^	*L. williamsii* F6_8S_P_1C^T^	*P. vandeheii*	*S. highlanderae*
Antibiotics	Multidrug resistance transporter, Bcr/CflA family	+	+	+	+	+	
Antibiotics	Multi antimicrobial extrusion protein (Na( +)/drug antiporter), MATE family of MDR efflux pumps			+	+	+	
Antibiotics	Acriflavin resistance protein			+	+	+	+
Antibiotics	Streptothricin acetyltransferase, Streptomyces lavendulae type					+	+
Antibiotics	Ribosome protection-type tetracycline resistance related proteins, group 2					+	+
Antibiotics	Fosfomycin resistance protein FosB					+	+
Antibiotics	Transcription regulator of multidrug efflux pump operon, TetR (AcrR) family					+	+
Antibiotics	Multidrug-efflux transporter, major facilitator superfamily (MFS) (TC 2.A.1)					+	
Antioxidant	PF00070 family, FAD-dependent NAD(P)-disulphide oxidoreductase	+	+	+	+		
β-lactamase	Metal-dependent hydrolases of the β-lactamase superfamily III	+				+	
Bile salt	Choloylglycine hydrolase (EC 3.5.1.24)					+	+
Cadmium	Cadmium-transporting ATPase (EC 3.6.3.3)					+	+
Cadmium	Cadmium efflux system accessory protein					+	+
Cadmium	Cadmium-transporting ATPase (EC 3.6.3.3)					+	+
Chromate	Chromate transport protein ChrA					+	+
Co-Zn-Cad	Cobalt-zinc-cadmium resistance protein CzcD					+	+
Copper	Copper resistance protein CopC	+	+	+	+	+	+
Copper	Copper resistance protein D	+	+	+	+	+	
Copper	Copper-translocating P-type ATPase (EC 3.6.3.4)	+	+	+	+		
Copper	Multicopper oxidase	+	+			+	
Copper	Copper chaperone		+	+	+		+
Copper	Cytoplasmic copper homeostasis protein CutC		+	+	+		
Copper	Copper-translocating P-type ATPase (EC 3.6.3.4)					+	
Heavy metal	DNA-binding heavy metal response regulator	+					+
Mercury	Mercuric ion reductase (EC 1.16.1.1)		+				
Mercury	Organomercurial lyase (EC 4.99.1.2)		+				+
Mercury	Mercuric resistance operon regulatory protein		+				+
Mg and Cobalt	Magnesium and cobalt efflux protein CorC		+	+	+		+
Zinc	Response regulator of zinc sigma-54-dependent two-component system					+	

Further analysis of these organisms from the ISS revealed that, on average, they possess 46 genes related to virulence, disease, and defense. Two mechanisms were predicted as the primarily function of these genes: resistance to antibiotics and toxic compounds, and invasion and intracellular resistance. *A. burdickii* IIF3SC-B10^T^, in particular, is the only species that harbors the sarcosine oxidase (EC 1.5.3.1) gene, which is involved in the osmotic stress response. On the other hand, *P. vandeheii* F6_3S_P_1C^T^ and *S. highlanderae* F6_3S_P_2^T^, both belonging to the phylum *Firmicutes*, possess a specific mechanism to respond to bacitracin-induced stress through the bceABRS four-component system. Notably, these two species also possess additional stress response mechanisms for periplasmic stress via the intramembrane protease RasP/YluC. While exploring other factors, it was observed that *A. burdickii* IIF3SC-B10^T^ does not possess any genes associated with motility. However, the other novel species have genes related to motility, including flagellar biosynthesis proteins. Among all the species, only *P. vandeheii* F6_3S_P_1C^T^ has the chemotaxis subsystem.

We conducted further investigations into the metabolic potential of these novel species and made some noteworthy observations. *A. burdickii* IIF3SC-B10^T^ was found to possess a significantly higher number of genes related to aromatic compound metabolism, while having fewer genes associated with iron metabolism compared to the other species characterized in this study. With the exception of *P. vandeheii* F6_3S_P_1C^T^, all the other species exhibited the capability to perform polyhydroxybutyrate metabolism. However, only *P. vandeheii* F6_3S_P_1C^T^, due to the presence of gamma-glutamyl transpeptidase (EC 2.3.2.2), can utilize glutathione as a sulfur source.

Considering the concerns regarding spore-forming bacteria and their resistance to sterilization processes in the ISS, we further explored the spore-forming capabilities of these novel species. As expected, a significant number of genes associated with dormancy and sporulation were predicted in *P. vandeheii* F6_3S_P_1C^T^ and *S. highlanderae* F6_3S_P_2^T^ genomes. Between them, *P. vandeheii* F6_3S_P_1C^T^ exhibited the highest number of 40 genes, primarily associated with spore germination and spore maturation processes. In contrast, the other species are non-spore forming and do not possess specific proteins associated with sporulation.

### Antimicrobial resistance properties of the novel species

In the isolated five novel species, we searched for antibiotic resistance genes against the CARD database^60^ and calculated the percentage identity with the reference sequences. Overall, these genomes showed potential resistance to seven drug classes, including rifamycin and tetracycline antibiotics. Interestingly, we found that all of these genomes contained genes from vancomycin resistance gene clusters with an identity ranging from 30.7% to 51.5%. Among other identified resistance genes, we discovered the presence of rifampicin monooxygenase (RIFMO) in *Leifsonia* species with a 63% match, which catalyzes the inactivation of the antibiotic rifampicin.

In *P. vandeheii* F6_3S_P_1C^T^, we identified a set of markers, including Llm 23S ribosomal RNA methyltransferase (LlmA_23S_CLI) and chloramphenicol acetyltransferase (CAT), which exhibited high sequence identities of 84.67% and 87.91%, respectively. LlmA_23S_CLI was originally detected in *Paenibacillus* sp. LC231, a strain isolated from Lechuguilla Cave, NM, USA^61^. Additionally, strain F6_3S_P_1C^T^ was found to possess two genes, qacG and qacJ, which are part of a small multidrug resistance efflux pump conferring resistance to quaternary ammonium compounds (QACs).

Furthermore, in *P. vandeheii* F6_3S_P_1C^T^ and *S. highlanderae* F6_3S_P_2^T^, we identified the presence of tetracycline-resistant ribosomal protection genes tetB(P) and tet(Q), respectively, with approximately 30% similarity. These provide resistance by preventing the binding of the antibiotic tetracycline to the bacterial ribosome. Moreover, these genomes also encode orthologues of the antibiotic-inactivating enzyme fosfomycin thiol transferase. The genomic mining predicted the presence of AMR genes but the confirmation of the phenotypic resistance requires further investigation.

### Production of secondary metabolites

To explore the potential for producing secondary metabolites in the newly discovered species, we utilized antiSMASH, a bioinformatics tool for predicting putative BGCs. This analysis revealed a total of 16 cluster types, including βlactone and type III polyketide synthase (T3PKS) clusters (Table 8). In *A. burdickii* IIF3SC-B10^T^, we identified a moderately matched thiopeptide antibiotic called TP-1161, known for its effectiveness against multidrug-resistant Gram-stain-positive bacteria and fungi^62^. Furthermore, in the isolated *Leifsonia* species, we found two well-known gene clusters: T3PKS-alkylresorcinol and NAPAA (non-α poly-amino acids) ε-Poly-l-lysine (ε-PL), both with a 100% match. Among the analyzed species, *Leifsonia* species shared a partially matched carotenoid BGC with *P. vandeheii* F6_3S_P_1C^T^. However, several unique cluster types, including cyclic-lactone-autoinducer, lanthipeptide, lassopeptide, NRP-metallophore, opine-like-metallophore, and proteusin, were identified exclusively in *P. vandeheii* F6_3S_P_1C^T^. Notably, within the F6_3S_P_1C^T^ strain, we also identified BGCs paeninodin (60%) and bacillopaline (100%). Lastly, *S. highlanderae* F6_3S_P_2^T^ exhibited one phosphonate, one T3PKS, and one terpene BGC, although these clusters have not yet been fully characterized.

**Table 8:** Bioactive gene clusters of novel ISS bacterial species.

Cluster Type	Known clusters	*Arthrobacter burdickii *IIF3SC-B10^T^	*Leifsonia virtsii* F6_8S_P_1A^T^	*Leifsonia williamsii *F6_8S_P_1B^T^	*Leifsonia williamsii *F6_8S_P_1C	*Paenibacillus vandeheii *F6_3S_P_1C^T^	*Sporosarcina highlanderae *F6_3S_P_2^T^
βlactone	Microansamycin	7					
Cyclic-lactone autoinducer						Unknown	
Furan				Unknown	Unknown		
Guanidinotides			Unknown				
Lanthipeptide-class-ii	Gramicidin S					15	
Lanthipeptide-class-iv							
Lassopeptide	Paeninodin					60	
Linaridin							
NAPAA	ε-Poly-l-lysine		100	100	100		
NRP-metallophore, NRPS	bacillibactin					100	
Opine-like-metallophore	Bacillopaline					100	
Phosphonate							Unknown
Proteusin						Unknown	
T3PKS							Unknown
	Alkylresorcinol		100	100	100		
	Corynecin I, II, and III					13	
	Meridamycin	5					
Terpene						Unknown	Unknown
	Carotenoid					33	
	TP-1161	25					
Terpene, βlactone	Carotenoid		28	28	28		

Each box depicts the percentage of similarity with the reported BGC. Unknown indicates a BGC was identified, but a percentage similarity was not calculated, since no known BGC was found to compare. Empty cells indicate that the BGC was not predicted in that genome.

## Discussion

New launch technology and new investment in human exploration of space by governments and private industry are leading to a revitalization of the idea of long-term space habitation. Missions to the moon are already underway, and missions to Mars are planned for the near future. Such missions will be measured in multiple years rather than in months and will have no or little resupply from Earth. In such cases, the microbiome of the space vessel or habitat will need to be monitored for multiple reasons: the spread of pathogens through the air or on surfaces which could infect humans^63^ or plants^64^ as well as the spread of antimicrobial resistance genes^65^, the health of human commensal microbiomes (and potential overgrowth of secondary pathogens), and the potential for biofouling of fluid lines or water supplies via microbial overgrowth^66^. Also, with no resupply from Earth there is no ability to gain access to Earth’s massive microbial biodiversity. Unless specific microbes are stocked as supplies before launch^1^, the microbes found on the spacecraft or habitat are the only ones which could be used for the many commercial purposes that microbes are used for on Earth: antibiotic or therapeutic discovery, manufacturing of drugs, food, and vitamins, plant growth enhancement, probiotics, etc. Biological *in-situ* resource utilization may also require bioremediation or bioconversion of raw, potentially toxic materials collected from moons, other planets, or asteroids/comets.

Whether or not the microbiome of a space habitat can be controlled and repurposed to this extent depends on a number of factors, including (1) whether the microbial diversity of such a space habitat would be sufficient to include all the traits desired for the many purposes listed above, (2) accurate detection and identification of already-known microbes and taxonomic placement of unidentified microbes, including whether shotgun metagenomic sequencing would detect the presence of problematic microbes from low biomass surfaces, and (3) characterization of potential phenotypic traits based on genomic predictions. Like the proverbial mustard seed, perhaps we inadvertently carry a planet’s worth of microbial diversity wherever we travel. The novel microbes described herein are not necessarily any more noteworthy than those which might be isolated from an office building on Earth [though they are likely far more resilient given the harsh conditions^67^ of the space environment], and yet each hosts significant potential for affecting human health^5^ or for use in assisting plant growth^68^, bioremediation or manufacturing, and offers a glimpse into the genetic and metabolic potential of the microbial diversity of the ISS^69^.

The most abundant cultivable microbes on ISS surfaces include common, well-studied human commensals such as *Staphylococcus, Rhodotorula, Penicillium,* and *Micrococcus* species. However, there are many more that have only been isolated once aboard the ISS and which are at very low abundance—potentially shed from individual astronauts, from experiments such as plant grow-ins, from new pieces of cargo, or from the vast microbial diversity of the human gut—which can be considered a part of the rare microbiome. Although individually rare, members of this community collectively play significant roles in ecosystem functioning and stability, including functional redundancy which enhances the resilience and stability of ecosystems by ensuring that multiple microbial species can perform essential ecological functions, such as nutrient cycling, decomposition, and symbiotic interactions. The novel bacterial species described during this study belong to rare microbial species since their incidence in the shotgun metagenomes was very low, and only the two *Leifsonia* species had sufficiently high breadth of coverage of mapped metagenomic reads from the ISS crew quarters to have been definitively identified using shotgun metagenomic sequencing without culturing as well.

Previous to using a WGS approach, the diversity of the ISS cultivable microbiome was significantly underestimated due to reliance on only 16S-based taxonomic identification. However, for many bacterial genera, 16S rRNA gene sequencing strategy fails to differentiate new species with significantly different phenotypic traits. For instance, there is 99.8% similarity between the 16S rRNA genes of *S. highlanderae* F6_3S_P_2^T^ and *S. thermotolerans* CCUG 53480^T^, with a mere 3 base pair substitutions. Without access to the whole genome, *S. highlanderae* would be categorized as *S. thermotolerans*, even though it is not a thermophile. Upon accessing all 93 *Sporosarcina* genomes from the NCBI database and generating an ANI heatmap (Supplemental Figure S2), it was evident that this clade contains at least five novel genera and 56 species which are yet to be described. This inference was based on ANI values of less than 70% for 14 *Sporosarcina* genomes, encompassing *S. highlanderae, S. thermotolerans,* and *S. luteola* and further emphasizes that the 16S rRNA gene on its own is not a reliable tool for differentiating among members of the *Sporosarcina* genus. Placing these 93 genomes into their phylogenetic affiliation require more study.

Upon examining 337 ISS bacterial genomes (plus six novel strains) belonging to 36 bacterial species (plus five novel species), it was observed that non-dominant, rare, and phylogenetically undescribed species predicted to produce natural products. As their genetic and phenotypic potential remains uncharacterized, exploration of the rare microbiome can lead to the discovery of novel bioactive compounds, enzymes, and metabolic pathways. Many of these rare microorganisms have untapped biotechnological potential, with applications in fields such as medicine, agriculture, industry, and environmental remediation. Studying the rare microbiome can uncover valuable resources for the development of new biotechnological tools and processes.

In the case of *A. burdickii* IIF3SC-B10^T^, we identified a moderately matched thiopeptide antibiotic called TP-1161, known for its efficacy against multidrug-resistant Gram-stain-positive bacteria and fungi^62^. *Leifsonia* species contain alkylresorcinol, which exhibits various activities including anticancer, anti-inflammatory, antimicrobial, antioxidant, and genotoxicity effects^70^. Additionally, alkylresorcinol plays a role in bacterial cyst formation during unfavorable environmental conditions^71^. On the other hand, ε-PL is responsible for antimicrobial activity against food spoilage and food-poisoning bacteria^72^.

In addition, *A. burdickii* and *P. vandeheii* also harbor metal-dependent β-lactamase superfamily-I and III proteins, which are known for their involvement in the hydrolysis of β-lactam antibiotics^73^. This enzyme family plays a significant role in conferring resistance to β-lactam antibiotics, including penicillins and cephalosporins. Furthermore, multidrug resistance efflux pumps such as the acriflavine resistance protein and Multidrug And Toxic Compound Extrusion (MATE) family of Multidrug Resistance (MDR) efflux pumps were found to be present in *Leifsonia* species and spore-formers. These efflux pumps contribute to bacterial resistance by actively pumping out a wide range of antimicrobial compounds from the cell, including antibiotics and toxic compounds, thereby reducing their intracellular concentrations and promoting bacterial survival. The acriflavin resistance protein (AcrA) is a crucial component of the AcrAB-TolC efflux pump, which confers resistance to acriflavin and other antimicrobial compounds. Its role in antibiotic resistance, multidrug efflux, intracellular homeostasis, biofilm formation, and potentially bacterial virulence underscores its significance in bacterial survival and adaptation. Understanding AcrA's function may aid in the development of strategies to combat antibiotic resistance and improve therapeutic approaches against multidrug-resistant bacterial infections. Given its involvement in antibiotic resistance and multidrug efflux, AcrA has emerged as a potential target for the development of novel antimicrobial agents^74^. By inhibiting the function of AcrA or other components of the AcrAB-TolC efflux pump, it may be possible to overcome bacterial resistance and enhance the effectiveness of existing antibiotics.

Identification of fosfomycin resistance protein (fosB) in both *Paenibacillus* and *Sporosarcina* genomes in this study is crucial for effective infection control measures and the development of strategies to combat the spread of antibiotic resistance. It has been reported that fosB is significant due to its impact on the treatment of bacterial infections, the emergence of multidrug resistance, the potential for horizontal gene transfer, and its implications for public health^75^.

Streptothricin acetyltransferase was present in both *Paenibacillus* and *Sporosarcina* genomes and it was reported that streptothricin is a valuable antibiotic with broad-spectrum activity against microorganisms and can help reduce crop losses and increase agricultural productivity^76^. Its significance extends beyond its direct antimicrobial properties, finding applications in research, agriculture, and drug development. Understanding streptothricin's mode of action and resistance mechanisms contributes to our knowledge of antibiotics and aids in the development of novel strategies to combat bacterial infections. Streptothricin has shown efficacy in agricultural practices, particularly in plant and fungal disease management^76^.

The ribosome protection-type tetracycline resistance-related proteins, group 2, are crucial determinants of resistance to tetracycline antibiotics in both Gram-stain-negative and positive microbes^77^. Their ability to protect ribosomes from the inhibitory effects of tetracycline enables microbial survival and growth in the presence of the antibiotic. The presence of these proteins in spore-forming novel species during this study and not in non-spore-forming bacteria needs further research.

Choloylglycine hydrolase plays a critical role in bile acid metabolism, enterohepatic circulation, and the regulation of the bile acid pool^78^ and predicted only in the *S. highlanderae* genome. Its activity influences the composition and function of the gut microbiota and has implications for host health and disease^79^. Understanding the significance of this enzyme provides insights into bile acid metabolism disorders^80^ and potential therapeutic approaches for related conditions.

Multiple genes for bioremediation of toxic material, enhanced plant growth, and survival in extreme conditions are predicted in the genomes of these novel species. Except for *P. vandeheii* F6_3S_P_1C^T^, all the other species possess the capability to perform polyhydroxybutyrate metabolism. However, *P. vandeheii* F6_3S_P_1C^T^ stands out as it can utilize glutathione as a sulfur source, due to the presence of gamma-glutamyl transpeptidase (EC 2.3.2.2)^81^.

Within the *P. vandeheii* F6_3S_P_1C^T^ strain, we identified paeninodin (60%) and bacillopaline (100%), which were originally characterized in *Paenibacillus dendritiformis* C454^82^ and *Paenibacillus mucilaginosus* KNP414^83^, respectively. Furthermore, this strain harbors a siderophore gene cluster (100%) responsible for producing the antimycobacterial agent bacillibactin^84^. The siderophore cluster also facilitates the conversion of iron from Fe3+ to Fe2+, which is a more usable form for the microbe and its plant host under iron-limited conditions. Siderophore synthesis gene clusters are rare in *Paenibacillus* species and are believed to have been acquired through horizontal gene transfer events^85^.

Genome mining resulted in identifying key functional genes of the novel species described in this study are listed in Table 7. Among all the novel species, mining of *A. burdickii* genome shows metal-dependent hydrolases of the β-lactamase superfamily III (MBL-III) enzyme. The MBL-III enzymes have shown potential in bioremediation processes aimed at the degradation of environmental pollutants^86^. The ability of MBL-III enzymes to degrade diverse chemical compounds, including pesticides, herbicides, and aromatic compounds, makes them valuable tools in the cleanup of contaminated environments^87^. These MBL-III enzymes can contribute to the removal and detoxification of pollutants, reducing their impact on ecosystems. Similarly, only in the genome of *A. burdickii*, DNA-binding heavy metal response regulator is predicted as shown in *Arthrobacter* sp. H-02-3^88^ which plays a crucial role in cellular responses to heavy metal exposure, including detoxification, metal homeostasis, stress response, and environmental adaptation.

In the genome of *L. virtsii*, organomercurial lyase is predicted which is reported to play a crucial role in the detoxification of organomercurial compounds, contributing to the protection of organisms and ecosystems by actinobacteria^88^. Its significance lies in the detoxification of toxic organomercurials, environmental protection, potential applications in bioremediation, insights into enzyme mechanisms, and biotechnological applications. Understanding and harnessing the capabilities of organomercurial lyase can aid in addressing mercury pollution and developing sustainable solutions for environmental and human health challenges^88^.

The magnesium and cobalt efflux protein (CorC) plays a significant role in maintaining metal ion homeostasis, protecting against metal toxicity, facilitating adaptation to metal-rich environments, and contributing to bacterial stress response. Its activity is important for cellular functions and can also impact antibiotic resistance. The identification of CorC in only three *Leifsonia* genomes during this study, while not observed in other novel species, holds significant potential for enhancing our understanding of the mechanisms employed by actinobacterial group to regulate metal ions and adapt to diverse environmental conditions.

In summary, the rare microbiome is instrumental in maintaining ecosystem stability, adapting to environmental changes, facilitating ecological interactions, spurring biotechnological innovation, and bolstering conservation efforts. Investigations into, and preservation of, the rare microbiome enhance our understanding of microbial diversity and ecosystem dynamics, thereby contributing to the sustainable management of the ecosystems. Conservation strategies should consider the preservation of rare microorganisms, as their loss could precipitate cascading effects on ecosystem functioning and resilience. Our study of novel microbes and predicted bioactive compounds contributes to our understanding of the microbial ecosystem on the ISS and lays the groundwork for further investigation into the potential implications of these novel species for the health and well-being of the ISS crew, as well as future space missions. The presence of specific genes and proteins in these novel species underscores their adaptive capabilities and potential resistance mechanisms against a variety of environmental challenges, including exposure to antibiotics. A deeper understanding of the genetic composition and functional capabilities of these novel species provides valuable insights into their survival strategies and could contribute to the development of improved antimicrobial therapies and strategies to combat antibiotic resistance.

### Supplementary Information

Supplementary Information.

## Data Availability

The draft genome sequences of all the strains characterized in this study were deposited in NCBI under BioProject PRJNA935338 and their BioSample accessions are: SAMN33786427 (*A. burdickii* IIF3SC-B10^T^), SAMN33786433 (*L. virtsii* F6_8S_P_1A^T^) SAMN33786428 (*L. williamsii* F6_8S_P_1B^T^), SAMN33786431 (*P. vandeheii* F6_3S_P_1C^T^), SAMN33786432 (*S. highlanderae* F6_3S_P_2^T^), and SAMN34051999 (*L. williamsii* F6_8S_P_1C). The WGS accession numbers are given in the species description section and the genome versions described in this paper are the first versions.
