# Supplementary material for: Phylogenomics, phenotypic, and functional traits of five novel (Earth-derived) bacterial species isolated from the International Space Station and their prevalence in metagenomes
Source: Sci Rep. 2023 Nov 6;13:19207. doi: 10.1038/s41598-023-44172-w (PMC10628120; doi:10.1038/s41598-023-44172-w)
Supplement: Supplementary file 1 — Supplementary Information. [file 41598_2023_44172_MOESM1_ESM.docx]

**Supplemental Table S1**. Vitek based biochemical profiles of novel species isolated from ISS.

| Species |  | *Arthrobacter burdickii* | *Leifsonia virtsii* | *Leifsonia williamsii* | *Paenibacillus vandeheii* | *Sporosarcina highlanderae* |
| --- | --- | --- | --- | --- | --- | --- |
| Vitek ID |  | *Kocuria rosea* | *Unidentified* | *Unidentified* | *Leuconostoc pseudomesenteroides* | *Alloiococcus otitis* |
| Strain # |  | IIF3SC-B10 | F6_8S_P_1C | F6_8S_P_1B | F6_3S_P_1C | F6_3S_P_2 |
| D-amygdalin | AMY | 0 | 0 | 1 | 0 | 0 |
| Phosphatidylinositol phospholipase C | PIPLC | 0 | 0 | 0 | 0 | 0 |
| D-xylose | dXYL | 0 | 0 | 1 | 0 | 0 |
| Arginine dihydrolase 1 | ADH1 | 0 | 0 | 1 | 0 | 0 |
| Beta-galactosidase | BGAL | 0 | 0 | 1 | 1 | 0 |
| Alpha-glucosidase | AGLU | 0 | 0 | 1 | 0 | 0 |
| Ala-Phe-Pro Arylamidase | APPA | 0 | 0 | 0 | 0 | 0 |
| Cyclodextrin | CDEX | 0 | 0 | 0 | 0 | 0 |
| L-aspartate arylamidase | AspA | 0 | 0 | 0 | 0 | 0 |
| Beta galactopiranosidase | BGAR | 0 | 0 | 0 | 1 | 0 |
| Alpha-mannosidase | AMAN | 0 | 0 | 0 | 0 | 0 |
| Phosphatase | PHOS | 0 | 0 | 0 | 0 | 0 |
| Leucine arylamidase | LeuA | 1 | 1 | 1 | 0 | 0 |
| L-proline arylamidase | ProA | 0 | 0 | 1 | 0 | 0 |
| Beta-glucuronidase | BGURr | 0 | 0 | 0 | 0 | 0 |
| Alpha-galactosidase | AGAL | 0 | 0 | 0 | 1 | 0 |
| L-Pyrrolydonyl-arylamidase | PyrA | 0 | 0 | 0 | 0 | 0 |
| Beta-glucuronidase | BGUR | 0 | 0 | 0 | 0 | 0 |
| Alanine arylamidase | AlaA | 1 | 1 | 1 | 0 | 0 |
| Tyrosine arylamidase | TyrA | 0 | 1 | 1 | 0 | 1 |
| D-sorbitol | dSOR | 0 | 0 | 0 | 0 | 0 |
| Urease | URE | 0 | 0 | 0 | 0 | 0 |
| Polymixin B resistance | POLYB | 0 | 0 | 0 | 0 | 0 |
| D-Galactose | dGAL | 0 | 1 | 1 | 0 | 0 |
| D-ribose | dRIB | 0 | 0 | 0 | 0 | 0 |
| L-lactate alkalization | ILATk | 0 | 1 | 1 | 0 | 0 |
| lactose | LAC | 0 | 0 | 0 | 0 | 0 |
| N-acetyl-D-glucosamine | NAG | 0 | 0 | 0 | 0 | 0 |
| D-maltose | dMAL | 0 | 0 | 1 | 0 | 0 |
| Bacitracin resistance | BACI | 0 | 0 | 0 | 1 | 0 |
| Novobiocin resistance | NOVO | 0 | 0 | 0 | 0 | 0 |
| Growth in 6.5% NaCl | NC6.5 | 0 | 0 | 0 | 0 | 0 |
| D-mannitol | dMAN | 0 | 0 | 1 | 0 | 0 |
| D-mannose | dMNE | 0 | 0 | 1 | 0 | 0 |
| Methyl-B-D-glucopyranoside | MBdG | 0 | 0 | 0 | 0 | 0 |
| Pullulan | PUL | 0 | 0 | 0 | 0 | 0 |
| D-raffinose | dRAF | 0 | 0 | 0 | 1 | 0 |
| O/129 resistance (comp. vibrio) | O129R | 0 | 0 | 0 | 0 | 0 |
| Salicin | SAL | 0 | 0 | 1 | 1 | 0 |
| Saccharose/sucrose | SAC | 0 | 0 | 1 | 1 | 0 |
| D-trehalose | dTRE | 0 | 1 | 1 | 1 | 0 |
| Agrinine dihydrolase 2 | ADH2s | 0 | 0 | 0 | 0 | 0 |
| Optochin resistance | OPTO | 0 | 0 | 0 | 1 | 0 |

**Supplemental Table S2.** BioLog-based biochemical profiles of novel species isolated from the ISS.

| Species | *Arthrobacter burdickii* | *Leifsonia virtsii* | *Leifsonia williamsii* | *Paenibacillus vandeheii* | *Sporosarcina highlanderae* |
| --- | --- | --- | --- | --- | --- |
| Strain # | IIF3SC-B10 | F6_8S_P_1C | F6_8S_P_1B | F6_3S_P_1C | F6_3S_P_2 |
| Negative control | 0 | 0 | 0 | 0 | 0 |
| Dextrin | 1 | 1 | 1 | 1 | 0 |
| D-maltose | 0 | 1 | 1 | 1 | 0 |
| D-trehalose | 0 | 1 | 1 | 1 | 0 |
| D-cellobiose | 0 | 1 | 1 | 1 | 0 |
| Gentiobiose | 1 | 1 | 1 | 1 | 0 |
| Sucrose | 0 | 1 | 1 | 1 | 0 |
| D-turanose | 0 | 1 | 1 | 1 | 0 |
| Stachyose | 1 | 1 | 1 | 1 | 1 |
| Positive control | 1 | 1 | 1 | 1 | 1 |
| pH6 | 1 | 1 | 1 | 1 | 0 |
| pH5 | 0 | 1 | 1 | 1 | 0 |
| Raffinose | 1 | 1 | 0 | 1 | 1 |
| α-D-Lactose | 1 | 1 | 1 | 1 | 1 |
| D-melibiose | 1 | 1 | 1 | 1 | 0 |
| β-Methyl-D-Glucoside | 1 | 1 | 1 | 1 | 0 |
| D-salicin | 0 | 1 | 1 | 1 | 0 |
| N-acetyl-D-glucosamine | 1 | 1 | 1 | 1 | 0 |
| N-acetyl-β-D-mannosamine | 0 | 1 | 1 | 1 | 0 |
| N-acetyl-D-galactosamine | 0 | 1 | 1 | 0 | 0 |
| N-acetyl neuraminic acid | 1 | 1 | 0 | 0 | 0 |
| 1% NaCl | 1 | 1 | 1 | 1 | 0 |
| 4% NaCl | 0 | 1 | 1 | 0 | 0 |
| 8% NaCl | 0 | 1 | 1 | 0 | 0 |
| α-D-Glucose | 0 | 1 | 1 | 1 | 1 |
| D-Mannose | 1 | 1 | 1 | 1 | 1 |
| D-fructose | 1 | 1 | 1 | 1 | 0 |
| D-galactose | 1 | 1 | 1 | 1 | 0 |
| 3-methyl glucose | 1 | 1 | 1 | 1 | 0 |
| D-fucose | 1 | 1 | 1 | 1 | 0 |
| L-fucose | 1 | 1 | 1 | 1 | 0 |
| L-rhamnose | 0 | 1 | 1 | 1 | 0 |
| Inosine | 1 | 1 | 0 | 1 | 0 |
| 1% sodium lactate | 1 | 1 | 1 | 1 | 0 |
| Fusidic acid | 1 | 1 | 1 | 0 | 0 |
| D-serine | 1 | 1 | 1 | 0 | 0 |
| D-sorbitol | 0 | 1 | 1 | 1 | 1 |
| D-mannitol | 0 | 1 | 1 | 1 | 0 |
| D-arabitol | 1 | 1 | 1 | 0 | 0 |
| myo-inositol | 1 | 1 | 0 | 0 | 0 |
| glycerol | 1 | 1 | 1 | 1 | 0 |
| D-glucose-6-phosphate | 1 | 1 | 1 | 1 | 0 |
| D-fructose-6-phosphate | 1 | 1 | 1 | 1 | 0 |
| D-aspartic acid | 1 | 1 | 0 | 0 | 0 |
| D-serine | 1 | 1 | 0 | 0 | 0 |
| Troleandomycin | 0 | 1 | 1 | 0 | 0 |
| Rifamycin SV | 1 | 1 | 1 | 0 | 0 |
| Minocycline | 1 | 1 | 1 | 0 | 0 |
| Gelatin | 1 | 1 | 1 | 0 | 1 |
| Glycyl-L-proline | 0 | 1 | 1 | 0 | 1 |
| L-alanine | 1 | 1 | 1 | 0 | 1 |
| L-arginine | 1 | 1 | 1 | 0 | 0 |
| L-aspartic acid | 1 | 1 | 0 | 0 | 0 |
| L-glutamic acid | 1 | 1 | 1 | 1 | 0 |
| L-histidine | 0 | 1 | 1 | 0 | 0 |
| L-pyroglutamic acid | 1 | 1 | 0 | 0 | 0 |
| L-serine | 1 | 1 | 1 | 0 | 0 |
| Lincomycin | 1 | 1 | 1 | 0 | 0 |
| Guanidine HCl | 0 | 1 | 1 | 1 | 0 |
| Niaproof 4 | 0 | 0 | 1 | 0 | 0 |
| Pectin | 1 | 1 | 1 | 1 | 1 |
| D-galacturonic acid | 1 | 1 | 1 | 1 | 1 |
| L-galactonic acid lactone | 1 | 1 | 1 | 1 | 0 |
| D-gluconic acid | 1 | 1 | 1 | 1 | 0 |
| D-glucuronic acid | 1 | 1 | 0 | 1 | 0 |
| Glucuronamide | 1 | 1 | 1 | 1 | 0 |
| Mucic acid | 1 | 1 | 0 | 0 | 0 |
| Quinic acid | 1 | 1 | 0 | 0 | 0 |
| D-saccharic acid | 1 | 1 | 0 | 0 | 0 |
| Vancomycin | 1 | 1 | 1 | 1 | 0 |
| Tetrazolium violet | 0 | 1 | 1 | 1 | 0 |
| Tetrazolium blue | 0 | 1 | 1 | 1 | 0 |
| p-hydroxy-phenylacetic acid | 0 | 1 | 1 | 0 | 0 |
| methyl puruvate | 0 | 1 | 0 | 1 | 0 |
| D-lactic acid methy ester | 0 | 1 | 0 | 0 | 1 |
| L-lactic acid | 1 | 1 | 1 | 0 | 0 |
| Citric Acid | 0 | 1 | 0 | 0 | 0 |
| α-keto-glutaric acid | 0 | 1 | 0 | 0 | 1 |
| D-malic acid | 1 | 1 | 1 | 0 | 0 |
| L-malic acid | 1 | 1 | 1 | 1 | 1 |
| Bromo-succinic acid | 0 | 0 | 0 | 0 | 0 |
| Nalidixic acid | 1 | 1 | 1 | 0 | 1 |
| Lithium chloride | 1 | 1 | 1 | 0 | 1 |
| Potassium tellurite | 0 | 1 | 1 | 1 | 0 |
| Tween 40 | 1 | 1 | 1 | 0 | 0 |
| γ-amino-butyric acid | 0 | 1 | 0 | 0 | 0 |
| α-hydroxy-butyric acid | 0 | 1 | 1 | 0 | 0 |
| β-hydroxy-D,L-butyric acid | 1 | 1 | 1 | 0 | 1 |
| α-keto-butyric acid | 1 | 0 | 0 | 0 | 0 |
| Acetoacetic acid | 0 | 1 | 1 | 1 | 1 |
| Propionic acid | 1 | 1 | 1 | 0 | 0 |
| Acetic acid | 1 | 1 | 1 | 1 | 1 |
| Formic acid | 1 | 1 | 0 | 0 | 1 |
| Aztreonam | 1 | 1 | 1 | 1 | 1 |
| Sodium butyrate | 1 | 1 | 1 | 1 | 1 |
| Sodium bromate | 0 | 1 | 0 | 1 | 0 |

**Supplemental Table S3.** Fatty acid profiles of the novel species isolated from ISS.

| Fatty acid name | *Arthrobacter burdickii* | *Leifsonia virtsii* | *Leifsonia williamsii* | *Paenibacillus vandeheii* | *Sporosarcina highlanderae* |
| --- | --- | --- | --- | --- | --- |
| 12:00 |  |  |  | 0.36 |  |
| 13:0 iso |  |  |  |  | 0.40 |
| 14:0 iso | 0.84 | 0.00 | 0.29 | 2.40 | **12.67** |
| 14:00 | 0.35 | 0.45 | 0.49 | 2.20 | 1.04 |
| 15:0 iso | 5.92 | 4.85 | 4.71 | 2.65 | **29.64** |
| 15:0 anteiso | **46.20** | 26.27 | **27.06** | **55.20** | **44.19** |
| 15:1 w6c | 0.24 |  |  |  |  |
| 16:1 w7c alcohol |  |  |  |  | 2.61 |
| 16:1 iso H | 4.62 |  |  |  |  |
| 16:0 iso | 7.72 | 12.15 | **11.76** | 9.06 | 3.62 |
| 16:1 w11c |  |  |  | 0.22 | 1.54 |
| 16:00 | 2.84 | 5.31 | 5.52 | **17.43** | 1.15 |
| 17:1 iso w10c |  |  |  |  | 0.28 |
| 17:1 anteiso w9c | 9.58 |  |  |  |  |
| 17:0 iso | 1.34 | 5.25 | 4.77 | 2.32 | 0.45 |
| 17:0 anteiso | **11.89** | 43.86 | **44.14** | 6.93 | 1.74 |
| 17:1 w8c | 0.19 |  |  |  |  |
| 17:0 cyclo | 0.29 |  |  |  |  |
| 17:00 |  | 1.13 | 1.27 |  |  |
| 18:0 iso | 0.53 | 0.14 |  |  |  |
| 18:1 w9c | 0.36 |  |  | 0.32 |  |
| 18:00 | 0.36 | 0.51 | 0.27 | 0.72 | 0.44 |
| Summed Feature 3 | 6.04 |  |  | 0.33 | 0.38 |
| Summed Feature 9 | 0.97 |  |  |  | 0.61 |

**Supplemental Table S4.** Annotated functional characteristics of the novel species isolated from the ISS.

| Subsystem Feature | *Arthrobacter burdickii IIF3SC-B10* | *Leifsonia virtsii F6_8S_P_1A* | *Leifsonia williamsii F6_8S_P_1B* | *Paenibacillus vandeheii F6_3S_P_1C* | *Sporosarcina highlanderae F6_3S_P_2* |
| --- | --- | --- | --- | --- | --- |
| Amino Acids and Derivatives | 289 | 260 | 252 | 282 | 224 |
| Carbohydrates | 255 | 242 | 199 | 329 | 169 |
| Cell Division and Cell Cycle |  |  |  | 5 | 5 |
| Cell Wall and Capsule | 21 | 32 | 34 | 54 | 39 |
| Cofactors, Vitamins, Prosthetic Groups, Pigments | 149 | 148 | 133 | 148 | 108 |
| DNA Metabolism | 71 | 73 | 61 | 86 | 68 |
| Dormancy and Sporulation | 1 | 1 | 1 | 40 | 18 |
| Fatty Acids, Lipids, and Isoprenoids | 63 | 61 | 51 | 35 | 103 |
| Iron acquisition and metabolism | 3 | 15 | 11 | 23 | 21 |
| Membrane Transport | 34 | 45 | 36 | 36 | 61 |
| Metabolism of Aromatic Compounds | 35 | 17 | 15 | 8 | 11 |
| Miscellaneous | 41 | 23 | 20 | 16 | 28 |
| Motility and Chemotaxis |  | 34 | 34 | 55 | 7 |
| Nitrogen Metabolism | 9 | 7 | 7 | 6 | 3 |
| Nucleosides and Nucleotides | 70 | 87 | 76 | 147 | 78 |
| Phages, Prophages, Transposable elements, Plasmids | 8 | 2 |  | 5 | 5 |
| Phosphorus Metabolism | 25 | 17 | 18 | 53 | 20 |
| Potassium metabolism | 5 | 8 | 8 | 5 | 2 |
| Protein Metabolism | 163 | 154 | 163 | 192 | 190 |
| Regulation and Cell signaling | 11 | 13 | 10 | 27 | 11 |
| Respiration | 45 | 54 | 40 | 42 | 42 |
| RNA Metabolism | 37 | 33 | 33 | 66 | 58 |
| Secondary Metabolism | 9 | 6 |  | 4 | 4 |
| Stress Response | 22 | 19 | 20 | 44 | 36 |
| Sulfur Metabolism | 5 | 7 | 6 | 20 | 10 |
| Virulence, Disease and Defense | 27 | 42 | 39 | 84 | 47 |

**Supplementary Figures**


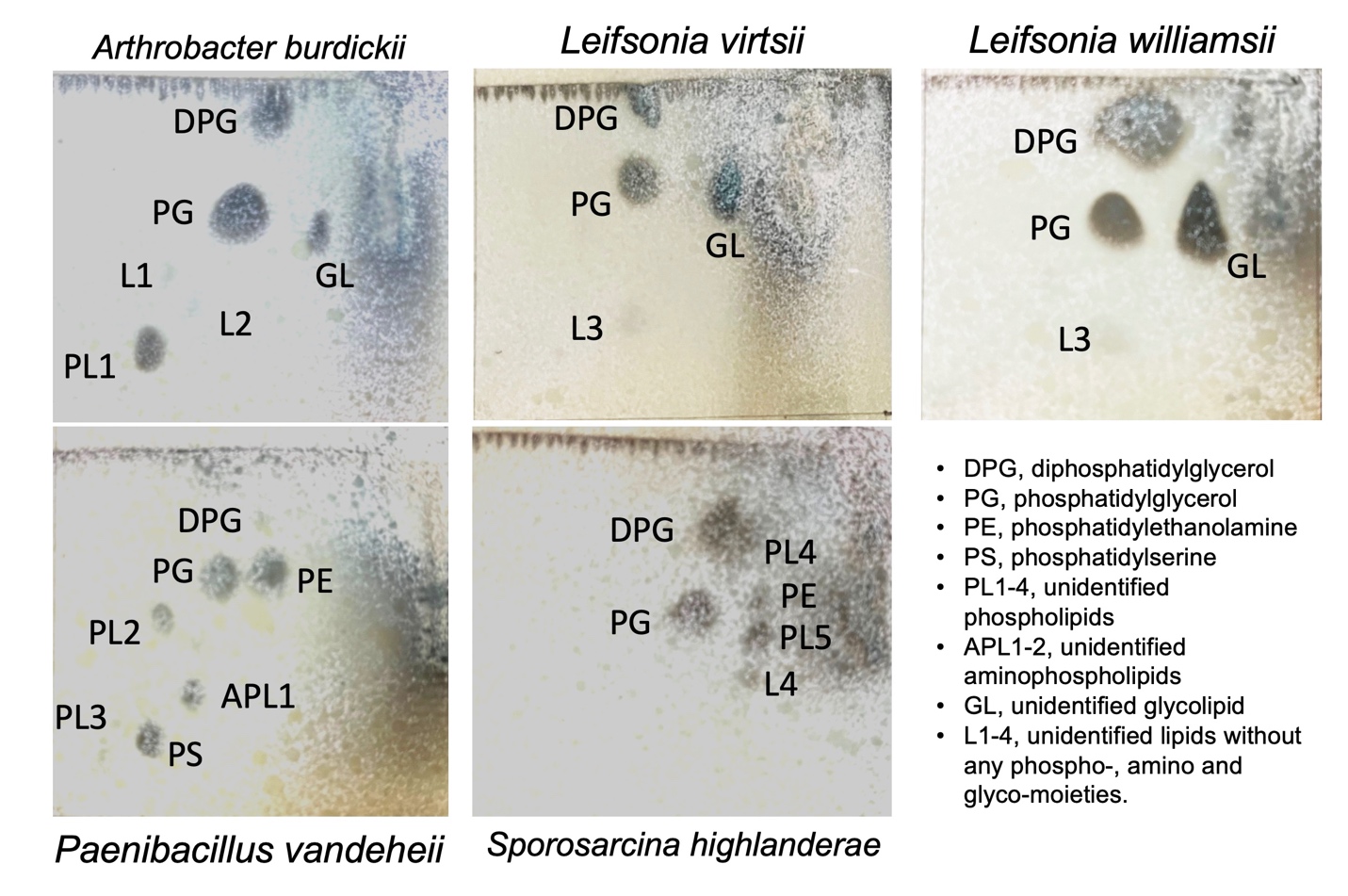


**Supplemental Figure S1.** Two-dimensional thin layer chromatographic polar lipid profiles of novel bacterial strain (a): *Arthrobacter burdickii* IIF3SC-B10^T^, (b): *Leifsonia virtsii* F6_8S_P_1A^T^, (c): *Leifsonia williamsii* F6_8S_P_1B^T^, (d): *Paenibacillus vandeheii* F6_3S_P_1C^T^, and (e): *Sporosarcina highlanderae* F6_3S_P_2 ^T^ isolated from the International Space Station. Lipids were stained with 10 % (w/v) ethanolic phosphomolybdic acid, 0.2 % (w/v) ninhydrin in butanol, Dittmer and Lester’s Zinzadze reagent and α-naphthol spray reagent to visualize total, amino, phsopho and glycolipids, respectively.


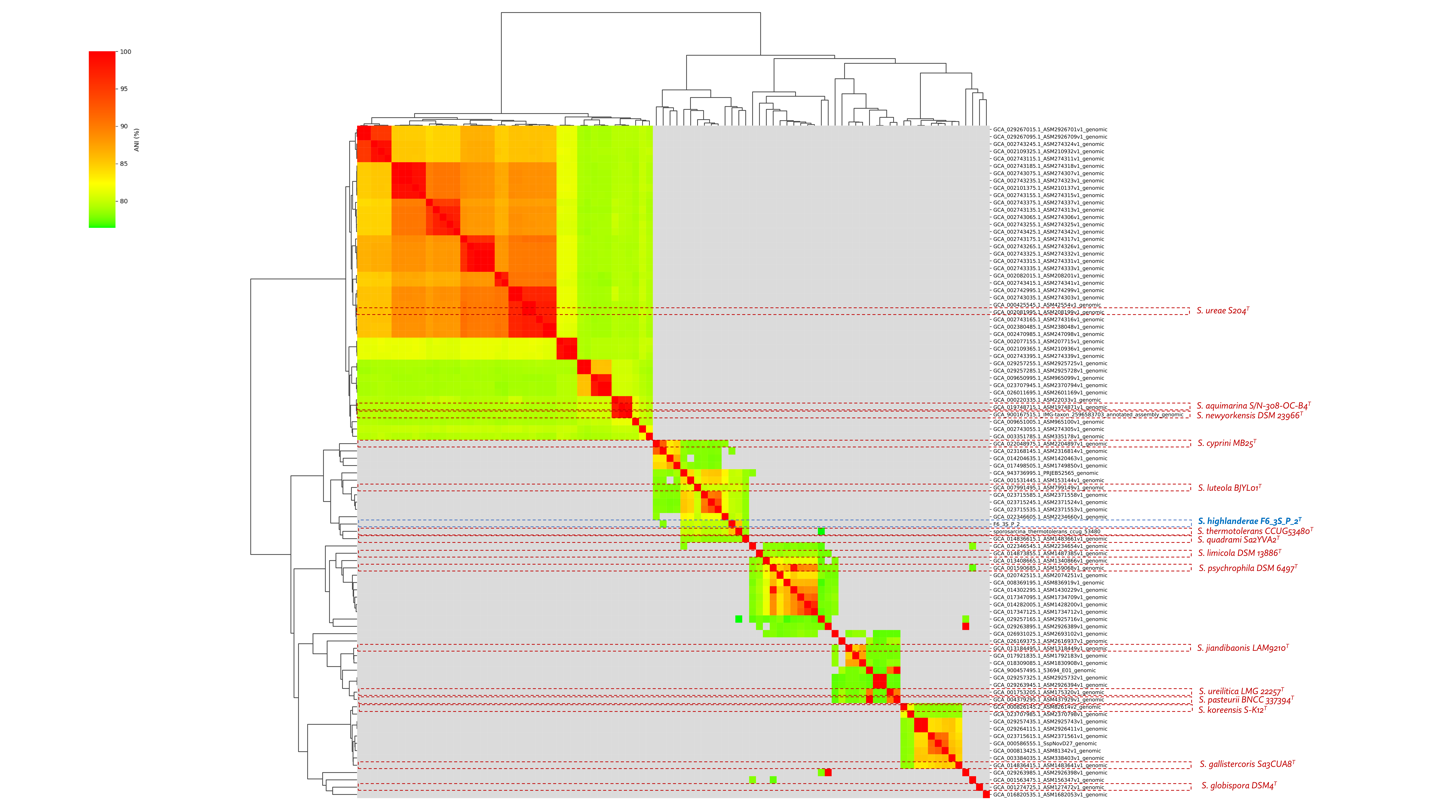


**Supplemental Figure S2:** All-vs-all average nucleotide identity (ANI) comparison of available *Sporosarcina* genomes on the GenBank database (n=93). The genomes of type strains of *Sporosarcina* species (n=15) are highlighted in red. Namely: *S. ureae* S204ᵀ, *S. aquimarina* S/N-308-OC-B4ᵀ, *S. newyorkensis* DSM 23966ᵀ, *S. cyprini* MB25ᵀ, *S. luteola* BYJL01ᵀ, *S. thermotolerans* CCUG53480ᵀ, *S. quadrami* Sa2YVA2ᵀ, *S. limicola* DSM 13866ᵀ, *S. psychropila* DSM 6497ᵀ, *S. jiandibaonis* LAM 9210ᵀ, *S. ureilitica* LMG 2257ᵀ, *S. pasteurii* BNCC 337394ᵀ, *S. koreensis* S-K12ᵀ, *S. gallistercoris* Sa3CUA8ᵀ, *S. globispora* DSM4ᵀ. The genome of *S. highlanderae* F6-3S-P2ᵀ is highlighted in blue.
